# Structural analysis of noncanonical translation initiation complexes

**DOI:** 10.1016/j.jbc.2024.107743

**Published:** 2024-09-01

**Authors:** Jacob M. Mattingly, Ha An Nguyen, Bappaditya Roy, Kurt Fredrick, Christine M. Dunham

**Affiliations:** 1 Department of Chemistry, Emory University, Atlanta, Georgia, USA; 2 Graduate Program in Biochemistry, Cell and Developmental Biology, Emory University, Atlanta, Georgia, USA; 3 Department of Microbiology and Center for RNA Biology, The Ohio State University, Columbus, Ohio, USA

**Keywords:** protein synthesis, translation initiation, tRNA^fMet^, A-minor motif, 16S rRNA

## Abstract

Translation initiation is a highly regulated, multi-step process that is critical for efficient and accurate protein synthesis. In bacteria, initiation begins when mRNA, initiation factors, and a dedicated initiator fMet-tRNA^fMet^ bind the small (30S) ribosomal subunit. Specific binding of fMet-tRNA^fMet^ in the peptidyl (P) site is mediated by the inspection of the fMet moiety by initiation factor IF2 and of three conserved G-C base pairs in the tRNA anticodon stem by the 30S head domain. Tandem A-minor interactions form between 16S ribosomal RNA nucleotides A1339 and G1338 and tRNA base pairs G30-C40 and G29-C41, respectively. Swapping the G30-C40 pair of tRNA^fMet^ with C-G (called tRNA^fMet^ M1) reduces discrimination against the noncanonical start codon CUG *in vitro*, suggesting crosstalk between the gripping of the anticodon stem and recognition of the start codon. Here, we solved electron cryomicroscopy structures of *Escherichia coli* 70S initiation complexes containing the fMet-tRNA^fMet^ M1 variant paired to the noncanonical CUG start codon, in the presence or absence of IF2 and the non-hydrolyzable GTP analog GDPCP, alongside structures of 70S initiation complexes containing this tRNA^fMet^ variant paired to the canonical bacterial start codons AUG, GUG, and UUG. We find that the M1 mutation weakens A-minor interactions between tRNA^fMet^ and 16S nucleotides A1339 and G1338, with IF2 strengthening the interaction of G1338 with the tRNA minor groove. These structures suggest how even slight changes to the recognition of the fMet-tRNA^fMet^ anticodon stem by the ribosome can impact the start codon selection.

Protein synthesis is a highly dynamic and delicately coordinated process involving the ribosome, mRNA, tRNAs and many translation factors. In bacteria, the start of translation (initiation) is generally the rate-limiting step that determines the translation efficiency of an mRNA (1). There are two major steps in initiation. The first step is the assembly of a 30S initiation complex (30S IC), involving base-pairing of the dedicated initiator tRNA, *N*-formyl-methionyl-tRNA^fMet^ (fMet-tRNA^fMet^), with the mRNA start codon in the peptidyl (P) site, accompanied by three initiation factors (IF1, IF2, IF3) (Fig. 1*A*). The second step involves the recruitment of the 50S ribosomal subunit, GTP hydrolysis by IF2, and dissociation of all three IFs to form a 70S IC, ready for elongation (2). The IFs act synergistically to regulate both the kinetics and fidelity of these steps (reviewed in (3, 4)). The GTPase IF2 ensures the correct selection of fMet-tRNA^fMet^ through recognition of the *N*-formyl moiety on the methionyl group at the tRNA acceptor end. IF2 mediates recruitment of the 50S ribosomal subunit to form the 70S IC, and its subsequent dissociation is driven by GTP hydrolysis (5, 6, 7, 8, 9). IF3 prevents the premature joining of the 50S subunit and allows for accurate start codon selection, while IF1 augments the activities of IF2 and IF3 (10).

**Figure 1:**
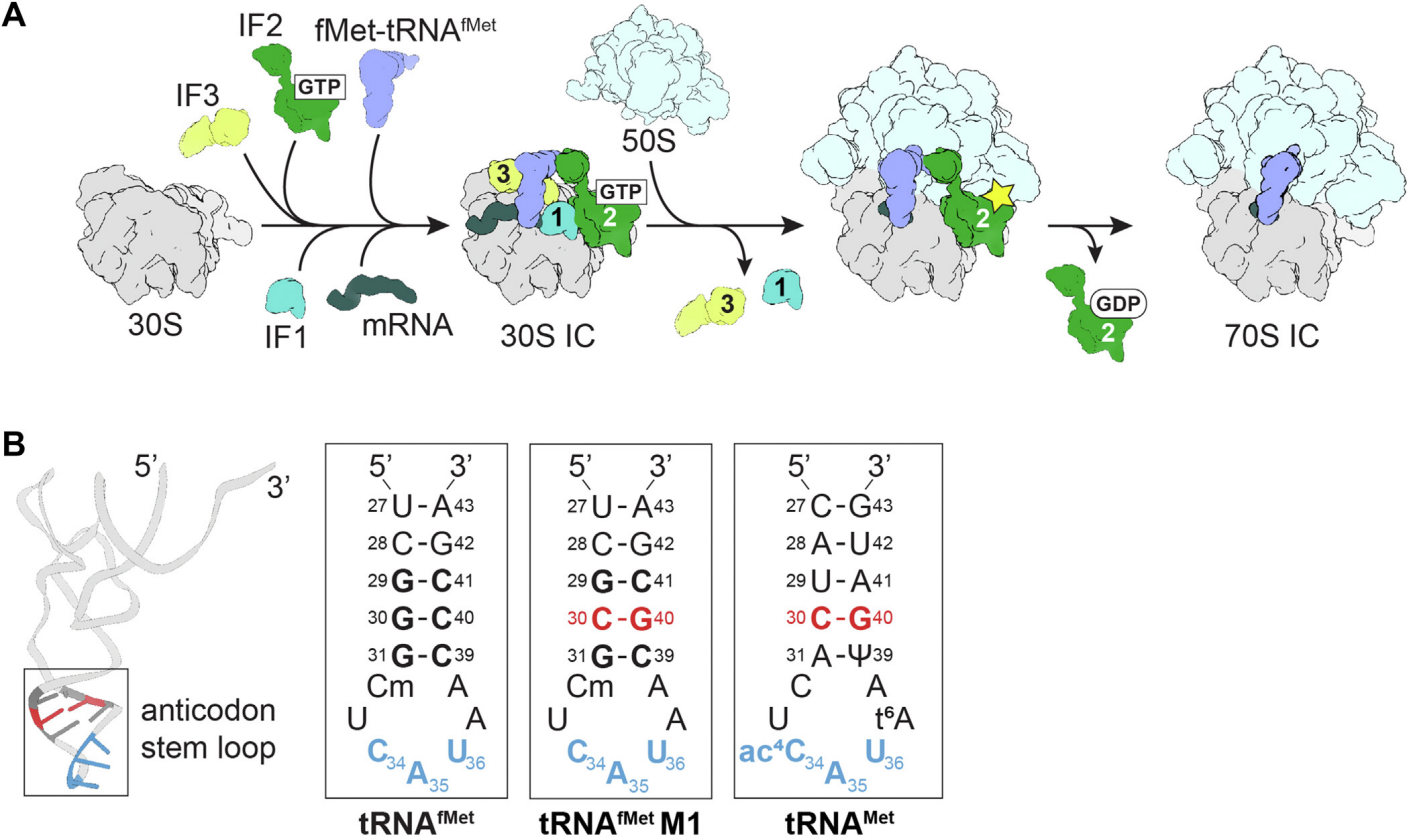
**tRNA**^**fMet**^**has unique features for translation initiation.***A*, translation initiation begins with initiation factors (IF1, IF2, IF3) binding to the 30S subunit in a defined order to guide fMet-tRNA^tRNA^ to the P site (called the 30S IC). 50S joins, GTP is hydrolyzed by IF2, and all factors are released. The resulting 70S IC is ready for the elongation phase. *B*, Mutation of the conserved G-C base pairs of the anticodon stem of tRNA^fMet^ M1 affect the ability of the ribosome to initiate translation on mRNA. Zoomed in view of the anticodon stem loop (ASL) of tRNA^fMet^ showing the anticodon (blue) and three conserved G-C base pairs (*bold*). The tRNA^fMet^ M1variant containing the C30-G40 mutation (*red*). The tRNA^fMet^ M1 30 to 40 base pair is identical to that of elongator tRNA^Met^.

In many bacteria, translation initiation entails recognition of a purine-rich mRNA region known as the Shine-Dalgarno (SD) sequence, located 7 to 10 nucleotides upstream of the AUG start codon of the mRNA (11). During initiation, the mRNA SD sequence pairs to the complementary anti-SD (ASD) sequence located at the 3′ end of the 16S ribosomal RNA (rRNA) of the 30S subunit. Formation of this SD-ASD helix helps to position the start codon in the P site (12, 13). The dedicated initiator tRNA^fMet^ contains distinctive features that specify its use in initiation rather than elongation (14). tRNA^fMet^ is aminoacylated with methionine and then formylated to yield *N*-formyl-methionyl-tRNA^fMet^ (fMet-tRNA^fMet^). Formylation depends on a unique C1•A72 base pair in the acceptor stem of tRNA^fMet^ and is critical for IF2 recognition (5, 15, 16). tRNA^fMet^ also has three conserved G-C base pairs (G29-C41, G30-C40, and G31-C39) in the anticodon stem that are important for P-site binding and efficient initiation *in vivo* (Fig. 1*B*) (17, 18, 19, 20, 21).

AUG, GUG, and UUG are considered canonical start codons in bacteria, accounting for 82%, 14%, and 4% of natural start codons, respectively, in a set of 69 representative genomes (22). The CAU anticodon sequence of tRNA^fMet^ forms a codon-anticodon pairing with the AUG start codon. First-position mismatches, *e.g.*, between tRNA^fMet^ and GUG and UUG start codons, are tolerated during initiation, in contrast to decoding that occurs in the A site (23, 24). In the A site, codon-anticodon base pairing is directly probed by 16S rRNA nucleotides for the selection of the correct tRNA. After peptidyl transfer and movement of the tRNA to the P site, the codon-anticodon helix of the tRNA is minimally inspected and instead, the 16S rRNA that surrounds the P-site tRNA grips its anticodon stem to ensure its correct positioning (13, 17, 25, 26). The gripping of the peptidyl-tRNA by the ribosome is critical for mRNA reading frame maintenance (27, 28). Interactions of the ribosomal P site with the tRNA include 16S rRNA nucleotides m^2^G966 and C1400 that pack beneath the third nucleotide of the anticodon (nucleotide 34; anticodon nucleotides are numbered 34, 35, 36) and A790, which contacts the tRNA backbone at nucleotide position 38. Conserved 16S nucleotides A1339 and G1338 of the 30S head domain form tandem type I and type II A-minor interactions with base pairs 30–40 and 29–41 of the tRNA in the P site during both initiation and elongation (18, 25, 29, 30). G30-C40 and G29-C41 are two of the three critical G-C base pairs in the anticodon stem of fMet-tRNA^fMet^ needed for efficient initiation (17, 18, 19) (Fig. 1*B*).

While GUG and UUG can act as alternate start codons, it is not clear why there is a sharp decrease in the frequency of usage of CUG as a start codon (0.024% of bacterial initiation codons) despite the tolerance of first-position codon-anticodon mismatches at the ribosomal P site (22). Start codon frequencies (AUG > GUG > UUG >> CUG) also correlate with translation efficiencies from each of the start codons compared to the AUG start codon, where GUG and UUG result in much higher levels of protein production (58.4% and 29.8% relative to AUG, respectively) than CUG (0.86% relative to AUG) (22). Both the thermodynamic and kinetic stability of fMet-tRNA^fMet^ binding to these start codons on the ribosome also depend on the identity of the first-position nucleotide (AUG > GUG ≈ UUG > CUG) (21). Interestingly, when the series of three G-C base pairs in the anticodon stem is disrupted, translation efficiency is reduced, with a single base pair change of G30-C40 to C30-G40 (called tRNA^fMet^ M1) decreasing translation by 20-fold *in vivo* (20) (Fig. 1*B*). The M1 variant loses the ability to discriminate against CUG, exhibiting P-site codon recognition properties similar to elongator tRNA^Met^. In contrast, wild-type tRNA^fMet^ has a marked preference for GUG and UUG over CUG (21). The C30-G40 base pair of the M1 variant is identical to that of elongator tRNA^Met^, suggesting a potential interplay between the tRNA anticodon stem and codon recognition in the 30S P site (21) (Fig. 1*B*). Recent studies using ribosome toeprint analysis, which measures the position of ribosomes on mRNAs, also indicate that complexes formed on model mRNAs with a CUG start codon show imprecise mRNA positioning, hypothesized to involve either a change in mRNA conformation in the ribosomal A site or frameshifting during translation initiation (21). This mRNA mispositioning is exacerbated by the tRNA^fMet^ M1 variant. IF2 restores normal mRNA positioning in these complexes, revealing a previously unknown quality control role for this initiation factor (21).

To determine how the ribosome interacts with the fMet-tRNA^fMet^ M1 variant, we solved five electron cryomicroscopy (cryo-EM) structures of *Escherichia coli* ribosome complexes containing fMet-tRNA^fMet^ M1: four complexes prepared without IF2 containing tRNA^fMet^ M1 paired with the AUG, GUG, UUG, or CUG start codons, and a fifth prepared with a CUG start codon and IF2 containing the non-hydrolyzable GTP analog GDPCP. We find that the G30-C40 to C30-G40 change weakens A-minor interactions formed by 16S rRNA nucleotides G1338 and A1339 in the absence of IF2, which could contribute to the apparent mRNA mispositioning reported previously. The A-minor interaction between 16S nucleotide G1338 and the tRNA anticodon stem appears to be strengthened in the presence of IF2, consistent with the ability of IF2 to restore mRNA positioning in analogous noncanonical initiation complexes. Finally, we find that 70S ICs prepared with tRNA^fMet^ M1 and a CUG start codon appear to occupy the normal reading frame and present their A-site mRNA codons in a conformation similar to functional 70S complexes, suggesting aberrant toeprint banding previously observed in 70S ICs containing tRNA^fMet^ M1 and a noncanonical CUG start codon likely arises from a change in mRNA conformation 3′ of the ribosomal A site.

## Results

### Variant M1 of tRNA^fMet^ exhibits weakened interactions with 16S rRNA nucleotides G1338 and A1339

**Figure 2:**
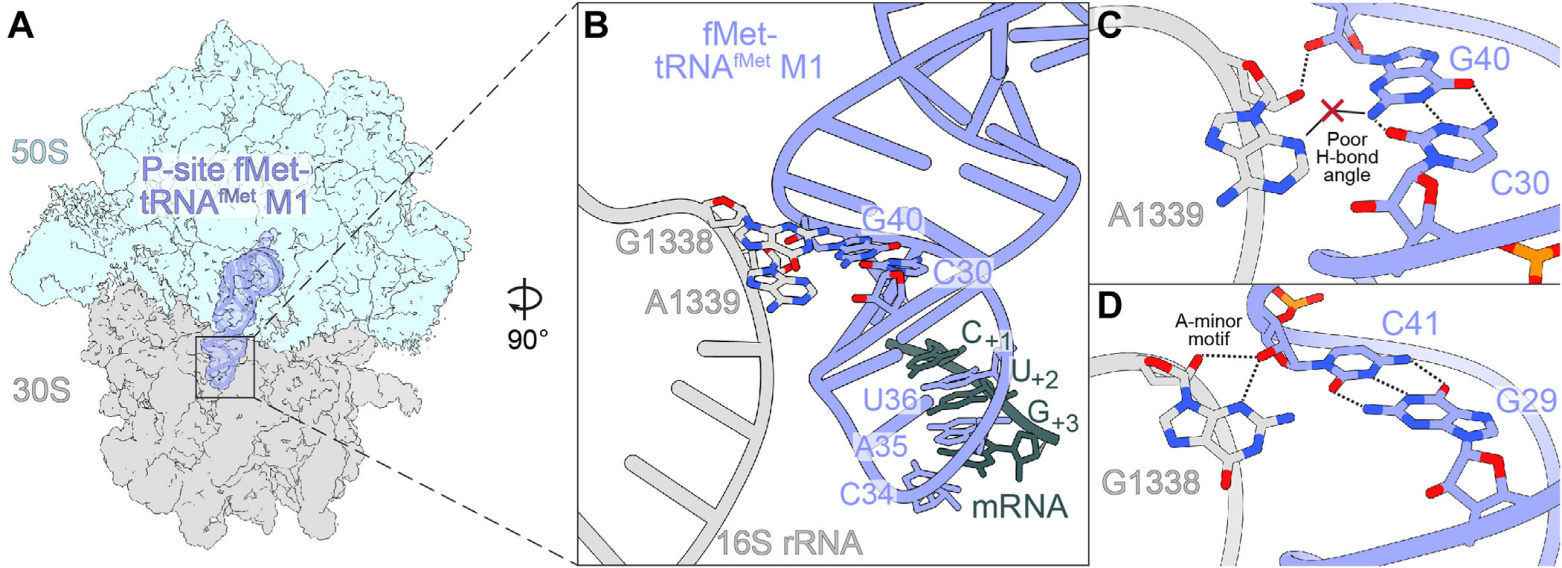
**One base pair change in the anticodon stem of tRNA**^**fMet**^**alters how the 16S rRNA recognizes initiator tRNA.***A*, Map of 70S ribosome complexes containing P-site fMet-tRNA^fMet^ with the M1 mutation (substitution of G-C with C-G at position 30–40). Map and model of tRNA are superimposed to enhance visibility. *B*, The tRNA^fMet^ M1 ASL interacts with 16S rRNA nucleotides G1338 and A1339. *C*, In the presence of the M1 mutation, the C30-G40 base pair forms weakened A-minor interactions with the base of conserved 16S rRNA nucleotide A1339. *D*, In the presence of the M1 mutation, tRNA^fMet^ nucleotide C41 forms A-minor interactions with the base of conserved 16S nucleotide G1338.

**Figure 3:**
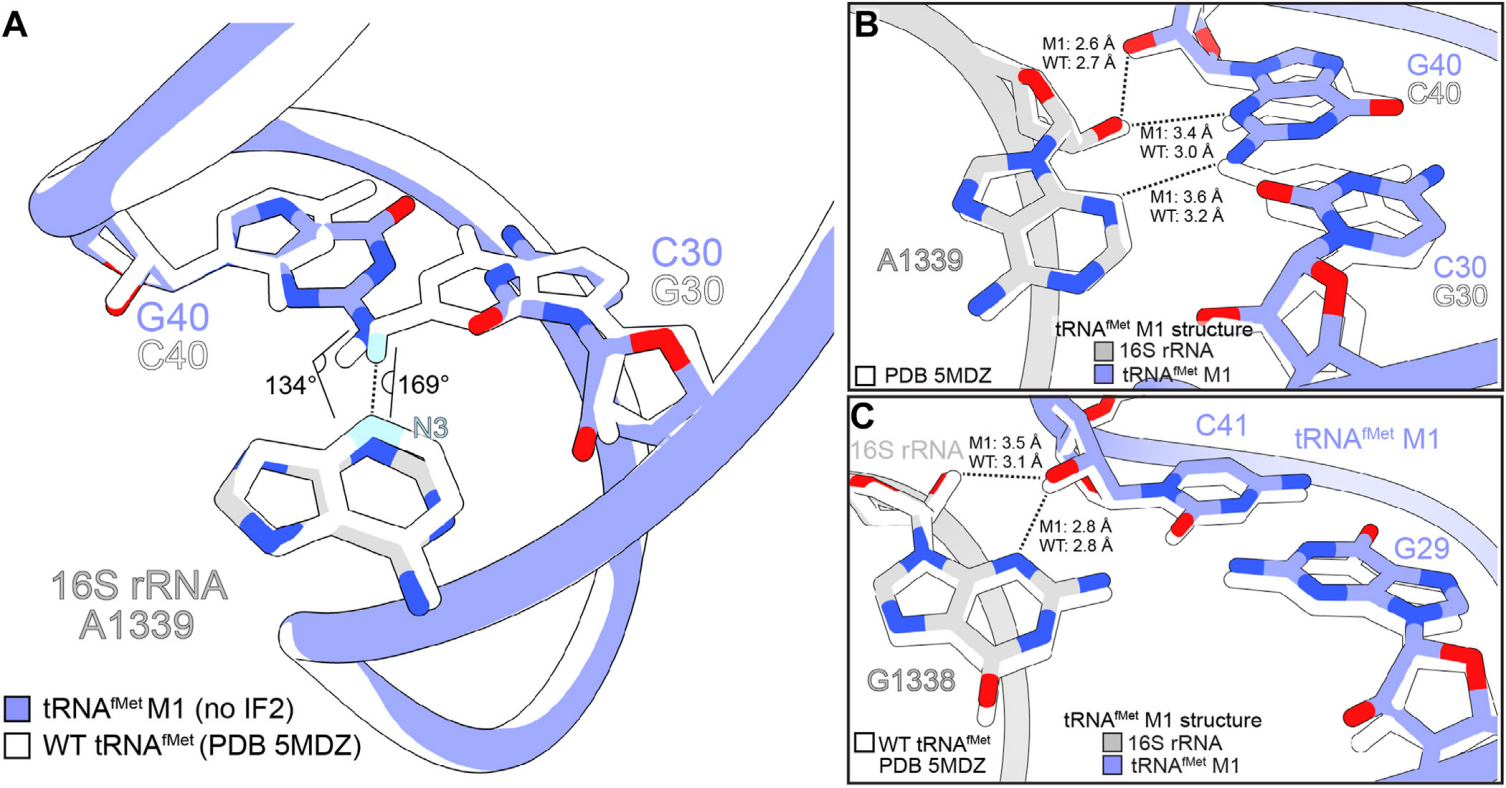
**The M1 mutation alters interactions with 16S rRNA nucleotides G1338 and A1339.***A*, in the absence of IF2, the tRNA^fMet^ M1 mutation tightens the hydrogen bonding angle between atom N3 of 16S rRNA nucleotide A1339 and hydrogen atom (denoted in *blue*) of the G of the 30 to 40 base pair, leading to poorer hydrogen bonding geometry compared to wild type tRNA^fMet^. *B*, compared to a structure containing wild type tRNA^fMet^ (PDB code 5MDZ), hydrogen bonds between A1339 and the tRNA^fMet^ M1 C30-G40 base pair are lengthened, reflecting weaker interaction. *C*, compared to wild-type tRNA^fMet^, the 2′-OH of tRNA^fMet^ M1 engages in more distant interactions with the G1338 2′-OH group, but its interaction with the G1338 base is unchanged.

In 70S ICs containing fMet-tRNA^fMet^ paired with an AUG start codon, 16S rRNA nucleotides A1339 and G1338 dock into the minor groove of the anticodon stem, making A-minor interactions with G30-C40 and G29-C41 (17, 18, 19). In the M1 variant, which contains C30-G40 rather than G30-C40, this recognition may be altered. To determine the structural effects of this base pair change, we determined the structures *of E. coli* 70S ICs containing the fMet-tRNA^fMet^ M1 variant in the P site paired to mRNA with the noncanonical start codon CUG (2.6 Å overall resolution) and the canonical bacterial start codons AUG (2.8 Å overall resolution), GUG (2.6 Å overall resolution), and UUG (2.6 Å overall resolution) (Fig. 2*A*; Figs. S1–S3, Tables S1–S4). As expected, fMet-tRNA^fMet^ nucleotides C30 and G40 form a Watson-Crick base pair, and this base pair change does not affect the pitch or width of the anticodon stem-loop compared to wild-type tRNA^fMet^ (Fig. S4*A*). Instead, the C30-G40 pair reorganizes its interactions with 16S rRNA nucleotides G1338 and A1339, which project into the tRNA minor groove (Fig. 2*B*). The type I A-minor motif interaction between the C30-G40 base pair and A1339 is weakened through the reduction of the hydrogen bond angle between the A1339 and G40 nucleobases. This interaction typically involves a nearly linear hydrogen bond between atom N3 of A1339 and the minor groove face of G30 in wild-type tRNA^fMet^, but the reorientation of hydrogen bond donor and acceptor groups in the tRNA^fMet^ minor groove due to the M1 mutation substantially reduces this hydrogen bond angle (WT: 169°; M1: 134°), weakening this interaction (Figs. 2*C* and 3*A*) (PDB code 5MDZ). Additionally, the interaction distance between the A1339 2′-OH and its nearest hydrogen bond acceptor on the position 40 base (atom O2 of C40 in wild-type tRNA^fMet^; atom N3 of G40 in tRNA^fMet^ M1) is increased by roughly 0.4 Å from 3.0 Å to 3.4 Å in complexes formed with tRNA^fMet^ M1 compared to those formed with WT tRNA^fMet^ (Fig. 3*B*) (31) (PDB code 5MDZ). Hydrogen bonding distances in the A1339-C30-G40 A-minor interaction are not substantially different in the presence of UUG, GUG, or AUG start codons compared to the CUG start codon, suggesting the strength of the interaction between tRNA^fMet^ M1 nucleotide G40 and 16S rRNA nucleotide A1339 is not dependent upon the identity of the mRNA codon first-position nucleotide (Fig. S5). Adjacent to this A-minor interaction is the wild type G29-C41 base pair, which forms a type II A-minor interaction with G1338. Specifically, the 2′-OH of tRNA^fMet^ M1 nucleotide C41 forms hydrogen bonds with the N3 atom and 2′-OH group of G1338 (Fig. 2*D*). Compared to a 70S ribosome complex prepared with wild-type tRNA^fMet^, the interaction distance between the G1338 and C41 2′-OH groups is increased from 3.1 Å to 3.5 Å, suggesting a slight weakening of hydrogen bonding, while the interaction distance between the G1338 base and C41 2′-OH group remains unchanged (2.8 Å) (Fig. 3*C*). Like the adjacent interaction of A1339 with the tRNA anticodon stem, the interaction between G1338 and C41 does not show meaningful differences in hydrogen bonding distances with canonical UUG, GUG, or AUG start codons compared to the non-canonical CUG start codon (Fig. S6). These results indicate that the simple swapping of G30-C40 to C30-G40 in the tRNA^fMet^ M1 variant weakens the ability of 16S rRNA nucleotides G1338 and A1339 to grip the tRNA at its minor groove through the disruption of important interactions with the anticodon stem.

### Weakened interactions between the tRNA^fMet^ M1 anticodon and the noncanonical CUG start codon

**Figure 4:**
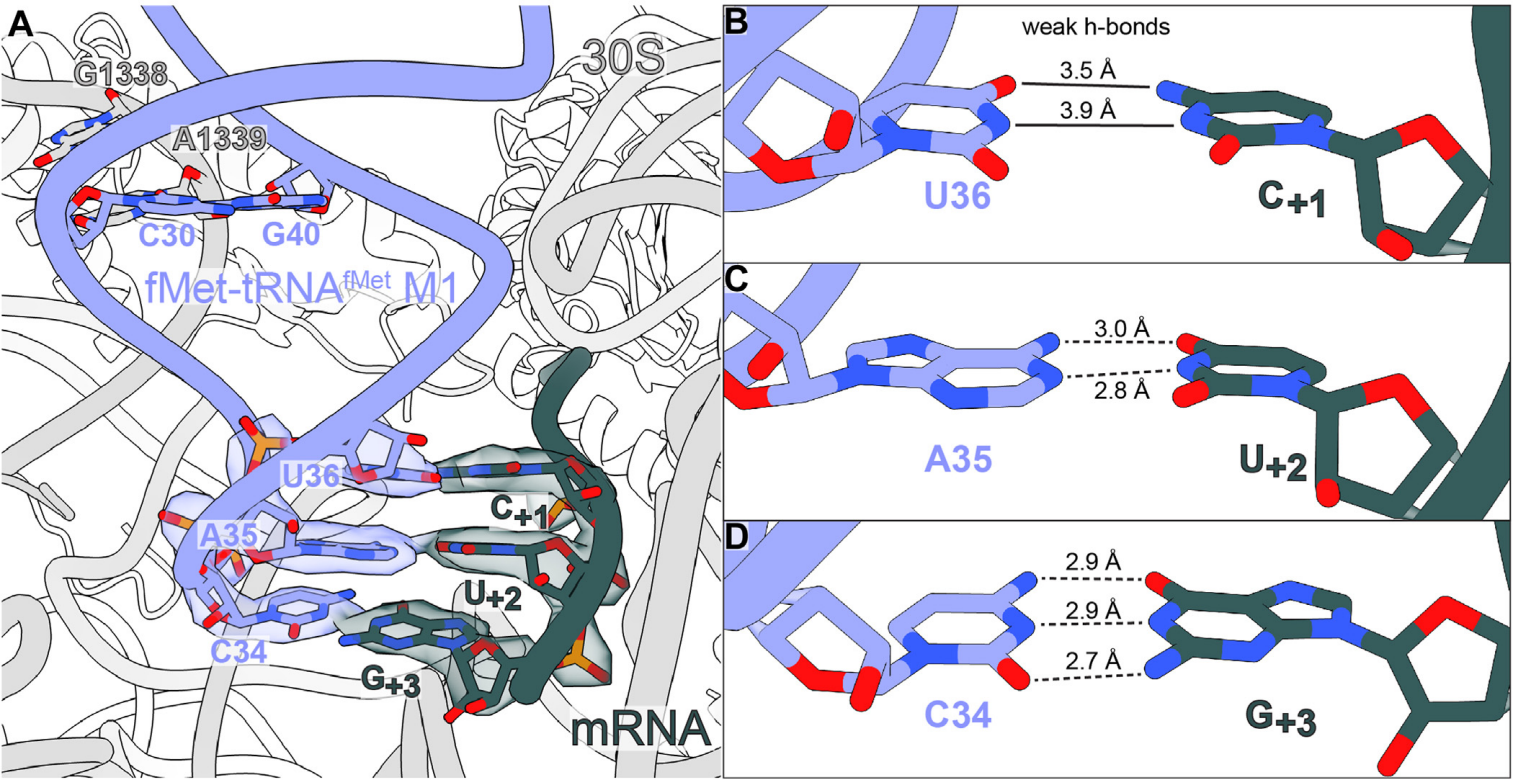
**Codon-anticodon interactions between tRNA**^**fMet**^**M1 and the CUG start codon in the absence of IF2.***A*, the CAU anticodon of tRNA^fMet^ M1 interacts with the noncanonical CUG start codon at the ribosomal P site (map threshold 0.21; map value range −0.877–1.41). *B*, Hydrogen-bond donors and acceptors are too distant for strong base pairing at the first position of the codon-anticodon interaction. *C*, base pairing at the second position of the codon-anticodon interaction displays typical hydrogen bonding distances. *D*, base pairing at the third position of the codon-anticodon interaction displays typical hydrogen bonding distances.

When tRNA^fMet^ M1 interacts with the CUG start codon, toeprint analysis indicates abnormal positioning of the mRNA, consistent with ribosome complexes appearing to occupy the 0 (normal), +1, and +2 reading frames, with the major product band consisting of apparent +2 frame complexes (21). This suggests a destabilization of mRNA which may allow the initiation complex to sample different positions. In our structure, we find that the CAU anticodon of tRNA^fMet^ M1 interacts with the CUG start codon in the normal reading frame, with map density in the tRNA exit (E) and P sites suggestive of 0-frame AAA and CUG codons, respectively (Fig. S7, *A* and *B*). However, the first-position codon-anticodon base pair between C_+1_ of the mRNA and U36 of fMet-tRNA^fMet^ M1 is weak (mRNA nucleotide numbering begins at the P site as +1, +2, and +3) (Fig. 4*A*). While their nucleobases are oriented with their Watson-Crick faces to pair, the hydrogen bond donor-acceptor distances observed are 3.5 Å and 3.9 Å, indicating only weak interactions between these nucleotides are possible (Fig. 4*B*). The second and third positions of the codon-anticodon interaction (U_+2_:A35 and G_+3_:C34, respectively) display hydrogen bonding distances and geometry consistent with typical Watson-Crick base pairing, ranging from 2.7 Å to 3.0 Å in donor-acceptor distances (Fig. 4, *C* and *D*). Because 70S ICs formed with tRNA^fMet^ M1 and canonical AUG, GUG, or UUG start codons display primarily 0 frame toeprint band formation, do not differ in their tRNA-ribosome interactions compared to CUG in our structures, and differ from CUG start codon complexes only in the identity of their P-site mRNA codon first-position nucleotides, it appears that mRNA mispositioning in ICs containing tRNA^fMet^ M1 and CUG start codons is mediated by differences in base pairing strength between CUG and the canonical start codons rather than by codon-specific differences in tRNA-ribosome interaction strength.

### 70S ICs containing tRNA^fMet^ M1 paired to a CUG start codon present their A-site codons in the 0 reading frame

To further investigate whether 70S complexes containing tRNA^fMet^ M1 interacting with a CUG start codon undergo +2 frameshifting during initiation in the absence of IF2, the cryo-EM sample containing mRNA with a CUG start codon and lacking IF2 was prepared in the presence of an excess of tRNA^IleX^, which decodes the AUA codon which would be presented at the A site in the event of a +2 frameshift. Notably, toeprint analysis studies have previously shown that apparent +2 frame toeprint bands comprise the major product in 70S ICs containing tRNA^fMet^ M1 and a CUG start codon (21). Therefore, if these 70S ICs undergo +2 frameshifting, a major population of particles should be observed which contains tRNA^IleX^ at the ribosomal A site. Interestingly, each cryo-EM dataset collected in the absence of IF2 contains a minor population of particles containing both P- and A-site tRNAs (CUG: 10.7% of 70S particles; UUG: 8.9% of 70S particles; GUG: 8.6% of 70S particles; AUG: 8.8% of 70S particles) (Figs. S1 and S2). In the datasets containing UUG, GUG, and AUG start codons, where only tRNA^fMet^ M1 was provided to the reactions, any A-site occupancy must be due to tRNA^fMet^ M1, representing a background level of A-site tRNA binding. The cryo-EM dataset prepared with a CUG start codon and excess tRNA^IleX^ displays only a slight enrichment in A-site tRNA-containing particles compared to UUG, GUG, or AUG datasets prepared without tRNA^IleX^, further suggesting that these complexes do not present +2 frame AUA codons at the ribosomal A site and primarily occupy the 0 reading frame.

An alternative explanation for previously observed toeprint band shifts in 70S ICs containing tRNA^fMet^ M1 paired to a CUG start codon is that the A-site codon is presented in an atypical, nonfunctional conformation which alters mRNA accessibility to reverse transcriptase downstream of the ribosome while not shifting the mRNA reading frame. This hypothesis is consistent with the observation that these 70S ICs are deficient in peptide bond formation activity, displaying a reduced rate and extent of dipeptide formation when the tRNA decoding the 0 frames A-site codon is added (21). The map quality for the mRNA in the A site of 70S ribosome complexes is typically poor in the absence of an A-site tRNA, but map density suggests three mRNA nucleotides occupy the A site in our structure in a conformation resembling that of a 70S complex competent for both initiation and subsequent elongation (31) (Fig. S7*C*) (PDB code 7K00). These results suggest that mRNA mispositioning observed in 70S ICs containing tRNA^fMet^ M1 paired with a CUG codon occurs downstream of the ribosomal A site rather than proceeding through frameshifting or aberrant presentation of the A-site mRNA codon.

### IF2 partially restores tRNA^fMet^ M1 interactions with the ribosomal P site

**Figure 5:**
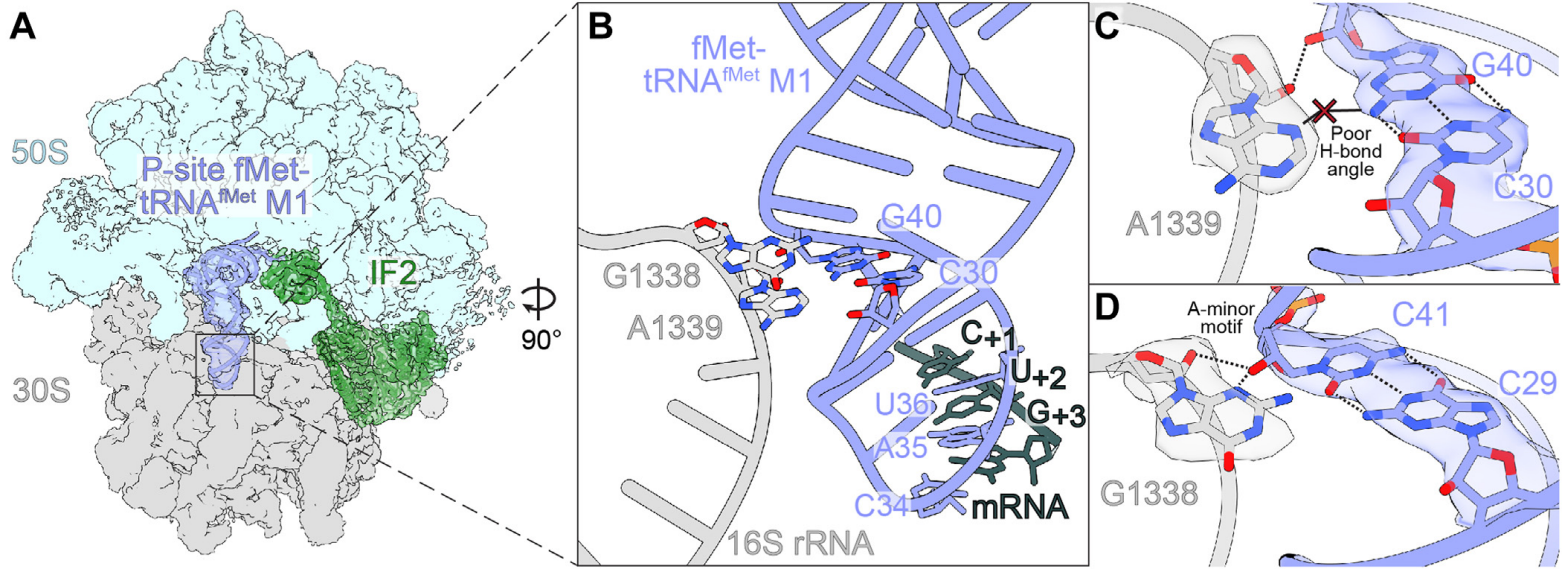
**IF2 partially restores gripping of tRNA**^**fMet**^**M1 by the 16S rRNA.***A*, map of 70S initiation complexes containing the IF2∙GDPCP∙fMet-tRNA^fMet^ M1 complex in the P site (tRNA and IF2 models and map segments superimposed for visibility). *B*, the tRNA^fMet^ M1 ASL containing the C30-G40 base pair interacts with 16S rRNA nucleotides A1339 and G1338 while maintaining base pairs with the CUG start codon. *C*, in the presence of IF2, the C30-G40 base pair is unable to form A-minor interactions with the nucleobase of A1339 (map threshold 5.0; map value range −11.3–28.2). *D*, in contrast, G1338 is oriented properly to form A-minor interactions with nucleotide C41 of the adjacent tRNA base pair (map threshold 4.6; map value range −11.3 to 28.2).

When the 50S ribosomal subunit is recruited during initiation, fMet-tRNA^fMet^ is constrained in the P/I orientation through its interaction with IF2 (“P” refers to the position on the 30S subunit and “I” refers to a unique initiation orientation on the 50S subunit, adjacent to the P site) (25). IF2 can suppress aberrant toeprint band formation in complexes containing tRNA^fMet^ M1 and a CUG start codon, suggesting that stabilization of tRNA^fMet^ M1 in the P/I orientation by IF2 may improve contacts between the 16S rRNA and tRNA^fMet^ anticodon stem (21). To determine the structural basis of IF2’s rescue activity, we determined a 2.6-Å cryo-EM structure of an *E. coli* 70S IC containing a P-site fMet-tRNA^fMet^ M1, mRNA with a P-site CUG start codon, and IF2 containing the non-hydrolyzable GTP analog GDPCP (Fig. 5*A*; Figs. S8, S9 and Table S5). As in our structure without IF2, we observe no effect on tRNA width or pitch due to the M1 mutation (Fig. S4*B*). Domain C1 of IF2 interacts with the GTPase center of the ribosome, and its C terminus spans the A and the P sites to directly bind the fMet moiety located in the 50S P site (32). In our structure, when IF2 is present, fMet-tRNA^fMet^ M1 continues to form interactions with the 16S rRNA nucleotides G1338 and A1339 (Fig. 5*B*). As in the absence of IF2, A1339 projects into the tRNA minor groove alongside the C30-G40 base pair, but poor hydrogen bonding geometry between G40 and A1339 precludes formation of strong type I A-minor interactions such as those observed with wild type tRNA^fMet^ (Figs. 5*C* and S10) (13). In contrast, IF2 strengthens interactions of G1338 with the adjacent G29-C41 base pair of tRNA^fMet^ M1. Compared to when IF2 is absent, we observe an approximately 2 Å displacement of the tRNA^fMet^ M1 anticodon stem loop (ASL) and the adjacent 16S rRNA loop containing G1338 and A1339 toward the ribosomal 30S head domain (Fig. S11*A*). We also observe that the position of G1338 in the tRNA minor groove is altered, with the G1338 base and 2′-OH group displaced 1.2 Å and 0.8 Å upward along the tRNA minor groove, respectively, and the 2′-OH of nucleotide C41 displaced 0.8 Å toward G1338 (relative to the C30-G40 base pair) compared to when IF2 is absent (Fig. S11*B*). This movement reduces hydrogen bond distances between G1338 and C41, permitting the N3 atom and 2′-OH of G1338 to form tighter A-minor interactions with the 2′-OH group of C41 (Figs. 5*D* and S11*B*). Together, these results indicate that IF2 constrains tRNA^fMet^ M1 in the P/I orientation and that this constraint is sufficient to strengthen interactions between the 30S head domain (in particular, G1338) and tRNA^fMet^ M1. This would, in turn, improve the ability of the ribosome to grip the P-site tRNA^fMet^ M1 and may contribute to the productive engagement of the CUG codon. These small movements that stabilize critical interactions could help explain how IF2 restores normal mRNA positioning in noncanonical complexes analyzed previously (21).

### IF2 does not influence pairing between the anticodon of tRNA^fMet^ M1 and the CUG start codon

**Figure 6:**
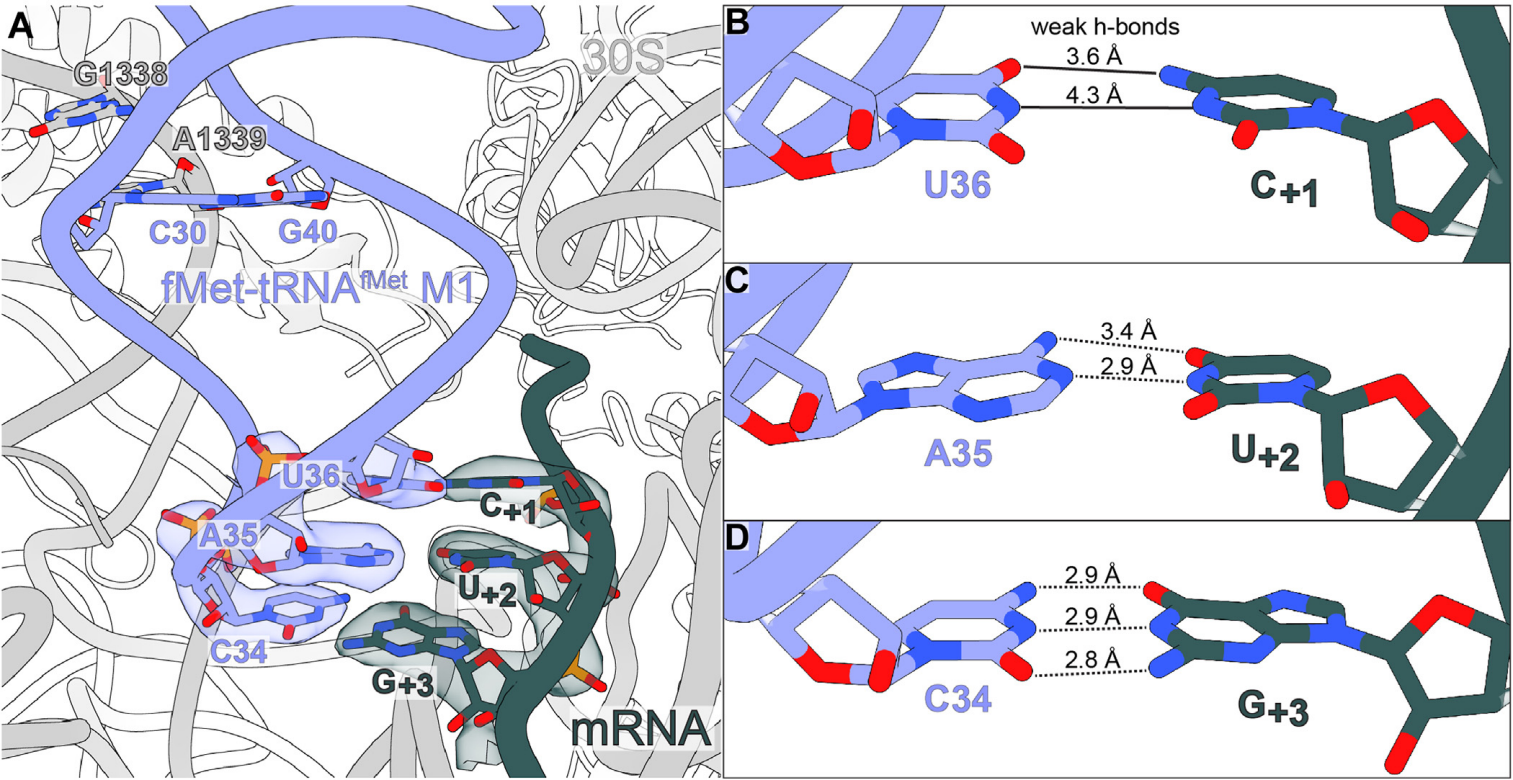
**IF2 does not appear to strengthen the interaction between the tRNA**^**fMet**^**M1 anticodon and the noncanonical CUG start codon.***A*, When IF2 is present, the CAU anticodon of tRNA^fMet^ M1 interacts with the P-site noncanonical CUG start codon (map threshold 4.5; map value range −11.3 to 28.2). *B*, IF2 interaction with tRNA^fMet^ M1 does not orient the first position codon-anticodon nucleotides closely enough for strong base pairing. *C*, base pairing at the second position of the codon-anticodon interaction displays typical hydrogen bonding distances. *D*, Base pairing at the third position of the codon-anticodon interaction displays typical hydrogen bonding distances.

We reasoned that if IF2 constrains the positioning of the tRNA at the CUG start codon such that the distance between the first position C_+1_ and anticodon nucleotide U36 is reduced, instability in mRNA positioning could be prevented through strengthening of the codon-anticodon interaction (Fig. 6*A*). However, in the presence of IF2, we find that there is no reduction of hydrogen bonding distance at the first position of the codon-anticodon interaction compared to when IF2 is absent (Fig. 6*B*). Additionally, as in the absence of IF2, the second and third positions of the codon-anticodon interaction maintain typical Watson-Crick base pairing distance and planarity (Fig. 6, *C* and *D*). Together, these results suggest that IF2’s ability to suppress apparent mRNA mispositioning does not seem to involve enhancement of codon-anticodon pairing strength in the ribosomal P site.

### 70S ICs containing tRNA^fMet^ M1 paired to a CUG start codon present their A-site codons in the 0 reading frame

Given the ability of IF2 to rescue normal mRNA positioning in toeprint analysis studies of 70S ICs containing tRNA^fMet^ M1 and CUG start codons and given that analogous 70S ICs formed without IF2 appear to occupy the 0 reading frame and present their A-site codons in a conformation resembling other elongation-competent 70S complexes (21), we predicted similar observations for 70S ICs containing tRNA^fMet^ M1, a CUG start codon, and IF2. As expected, these complexes display map density in the E and P sites consistent with AAA and CUG codons, respectively, and map density in the A site suggests the mRNA codon is presented similarly to 70S complexes productively engaging an A-site tRNA (31) (Fig. S7, *D*–*F*) (PDB code 7K00).

## Discussion

In this work, we sought to gain insight into initiation fidelity by determining structures of noncanonical complexes carrying the fMet-tRNA^fMet^ M1 variant paired with the noncanonical CUG start codon and canonical AUG, GUG, and UUG start codons (21). Changing the G-C base pair at position 30 to 40 of tRNA^fMet^ to C-G weakens the A-minor interactions formed between 16S rRNA nucleotides A1339 and G1338 and the tRNA minor groove, which manifests in a reduced ability to discriminate against CUG start codons compared to wild type tRNA^fMet^. While the change in the positions of G1338 and A1339 projecting into the tRNA minor groove is small, the functional implications are substantial given that these changes reduce discrimination by decreasing the stability of canonical GUG and UUG complexes by 10-fold but not further destabilizing CUG complexes relative to ICs formed with wild type tRNA^fMet^ (21).

The observation that tRNA^fMet^ M1 displays weakened A-minor interactions with the 16S rRNA is consistent with prior analysis of the thermodynamics and prevalence of A-minor motifs in structured RNAs (33). Type I A-minor motifs are strongest when the interaction consists of adenosine buried in the minor groove of a G-C base pair with the A- and C- containing strands antiparallel to one another, as is the case for the interaction of 16S nucleotide A1339 with the G30-C40 base pair in wild-type tRNA^fMet^. In the P4-P6 domain of the *Tetrahymena* group 1 intron, there is a roughly 25% decrease in the free energy of formation of type I A-minor motifs formed with G-C base pairs where the A- and G-containing RNA strands are antiparallel (relative to interactions with the A- and C-containing strands antiparallel). This example is similar to the interaction of 16S rRNA nucleotide A1339 with tRNA^fMet^ M1. The stabilizing impact of strong type I A-minor interactions may be further exemplified by the widespread nature of type I interactions with antiparallel A- and C- strands as seen in a phylogenetic analysis of 23S rRNA from *H. marismortui* and *T*. *thermophilus* (33). In this analysis, 89% of observed type I A-minor motifs consisted of G-C base pairs with antiparallel A- and C-containing strands (as observed with wild type tRNA^fMet^), while only 6% consisted of G-C pairs with antiparallel A- and G-containing strands (as observed with tRNA^fMet^ M1). This prevalence among highly structured RNAs is suggestive of the importance of strong type I A-minor interactions for structural stability. The thermodynamic stability of type I A-minor motif interactions between A1339 and wild type tRNA^fMet^ provides additional context for why weakening this interaction in the tRNA^fMet^ M1 variant disrupts normal translation initiation by loosening the ribosome’s grip on the tRNA.

Interestingly, *E. coli* ribosomes containing the 16S rRNA mutation G1338A, which strengthens the interaction of the 16S rRNA with the tRNA^fMet^ ASL minor groove, undergo spurious initiation at noncanonical start codons, including CUG, more frequently than wild-type ribosomes (19). Conversely, mutations of position A1339, which weaken the tRNA-ribosome interaction at this position, yield ribosomes with greatly reduced translation activity (17, 18). These results suggest that translation initiation may be fine-tuned for both efficiency and accuracy in a manner that depends on the strength of interactions of the initiator tRNA with both the mRNA codon and the ribosome. Excessively strong ribosome-tRNA interactions cause initiation at incorrectly selected start codons while weak ribosome-tRNA or tRNA-mRNA interactions, as observed for tRNA^fMet^ M1 initiating translation on the start codon CUG, cause inefficient initiation or nonproductive engagement of the start codon. Given the apparent lack of a direct effect of the first-position codon-anticodon interaction on the strength of tRNA-rRNA interactions between 16S nucleotides A1339 and G1338 and the tRNA minor groove, the ribosome may independently require sufficiently strong rRNA-tRNA and mRNA-tRNA interactions for productive initiation. Previous competition binding assays demonstrate that 30S and 70S ICs assembled with CUG start codons are less thermodynamically and kinetically stable than those formed with AUG, GUG, or UUG start codons irrespective of whether they contain wild-type tRNA^fMet^ or tRNA^fMet^ M1 (21); however, the discrepancy between the stability of ICs containing a CUG start codon and those containing a canonical start codon is smaller when they are formed with tRNA^fMet^ M1. This reduction in the discrepancy may help account for the reduction in the ability of tRNA^fMet^ M1 to discriminate against CUG start codons, which in previous experiments results from a reduction in preference for the canonical but near-cognate start codons GUG and UUG rather than an increase in preference for pairing with CUG, on which neither wild type tRNA^fMet^ nor the M1 variant efficiently initiate translation (21).

One previously observed consequence of the C30-G40 base pair change in tRNA^fMet^ M1 is mRNA mispositioning, as assessed by toeprint analysis (21). In these assays, initiation complexes containing the tRNA^fMet^ M1 variant and mRNA containing the noncanonical CUG start codon appear to occupy the 0 (normal), +1, and +2 reading frames, with the apparent +2 frame band forming the major product. This toeprint heterogeneity is only observed with tRNA^fMet^ M1 engaging a CUG codon, not with AUG, GUG, or UUG (21). The +1 or +2 frame would indicate a movement of the ribosome toward the 3′ end of the mRNA, presumably moving the C_+1_ or U_+2_ nucleotide into the E site with a change of the nucleotides that are positioned in the P site. Another interpretation is that the mRNA conformation in the ribosomal A site is altered in some way that exposes the next codon in a shifted downstream register without changing the codon presented in the P site (21). We conclude from our structure of 70S ICs containing tRNA^fMet^ M1 paired with the CUG start codon that tRNA^fMet^ M1 engages the CUG codon in the 0 reading frame, based on map density for the P-site mRNA codon and our observation that adding an excess of tRNA^IleX^, which decodes the +2 frame AUA codon, causes only a very slight enrichment in the proportion of picked 70S particles displaying tRNA occupancy at the ribosomal A site compared to AUG, GUG, and UUG start codon datasets prepared only with tRNA^fMet^ M1. Additionally, the conformation of the A-site mRNA codon appears similar to that observed in 70S complexes productively engaging tRNAs at the A site (31) (PDB code 7K00). Therefore, while prior biochemical assays show an apparent change in mRNA register or conformation at the P or A site, our structural data suggest that the mRNA is normal in the context of the P and A sites and that prior toeprint analysis assays are reporting on features of these ICs other than the mRNA reading frame or A-site codon conformation. Another possibility is that the mRNA path is altered 3′ of the A-site codon, giving rise to the shorter toeprints, although the mRNA was not resolved 3′ of the A site in the cryo-EM maps and thus this possibility cannot be evaluated from our structures alone.

Prior ribosome structural studies have focused on understanding single base pair mismatches between the codon and the anticodon at the P site in both initiation and elongation contexts (34, 35). Several of these elongation structures contain pyrimidine-pyrimidine mismatches at the P site and while the mismatched nucleotides present their Watson-Crick faces for pairing, observed hydrogen bond donor-acceptor distances are not within accepted ranges and are suggestive of weak interactions. For example, in a structure with a third position C_+3_-C34 mismatch, the closest donor-acceptor distance is 3.6 Å, and in a structure of a first-position U_+1_-U36 mismatch, the donor-acceptor distances are 4.0 Å and 4.5 Å (34, 35). In our 70S IC structures containing a first-position C_+1_-U36 mismatch, we observe hydrogen bond donor-acceptor distances of 3.5 Å and 3.9 Å, also suggesting weak interaction. Crucially, G-U and U-U base pairs, which are both tolerated at the first position of the codon-anticodon interaction during initiation, may both form two hydrogen bonds in studies of model RNA duplexes, while C-U pairs have been observed to form either one or two hydrogen bonds in model RNAs (36, 37). Additionally, it is known empirically that 30S and 70S ICs formed with CUG start codons are less stable than those formed with the canonical bacterial start codons AUG, GUG, and UUG (21). If base-pairing strength at the first position of the start codon is important for efficient translation initiation in addition to strong interactions between the initiator tRNA and 16S rRNA, this may help explain why CUG is not used as a common non-AUG start codon in bacteria, in contrast to GUG and UUG start codons (22).

IF2 imposes physical constraints on fMet-tRNA^fMet^ that appear to rescue mRNA positional instability in the context of CUG start codons and contribute to productive initiation (21). We find that IF2 adopts a normal open conformation with domain C2 recognizing the fMet moiety of the initiator tRNA (Fig. S11*A*). This recognition, and the subsequent constraining of tRNA^fMet^ in the P/I orientation by IF2, also appears to allosterically regulate how the 30S head domain nucleotide G1338 grips the anticodon stem of tRNA^fMet^ M1, strengthening the type II A-minor interaction between G1338 and tRNA^fMet^ nucleotide C41 by permitting G1338 to project more deeply into the tRNA minor groove. While it is not clear if reestablishing the type II A-minor contacts between G1338 and tRNA^fMet^ M1 in the presence of IF2 is itself directly responsible for stabilizing the mRNA placement, these findings are consistent with prior biochemical studies demonstrating that IF2 is sufficient to restore wild-type-like codon discrimination behavior in ICs formed with tRNA^fMet^ M1 and mRNA containing a CUG start codon (21).

## Experimental procedures

### Ribosome purification

Ribosomes were purified from *E. coli* MRE600 cells as previously described (38, 39). Briefly, cells were grown in lysogeny broth (LB) to OD_600_ 0.7 at 37 °C in a shaking incubator and then cooled on ice for 20 min. Cells were pelleted by centrifugation, washed with buffer 1 (10 mM HEPES-KOH, pH 7.6, 10 mM MgCl_2_, 1M NH_4_Cl, 6 mM β-mercaptoethanol (β-Me)) twice then resuspended in buffer 2 (buffer 1 with 100 mM NH_4_Cl) and lysed using the Emulsiflex-C5 high-pressure homogenizer (Avestin). The lysate was clarified by centrifugation at 27,000*g* for 30 min, followed by another spin at 42,000*g* for 17 h to pellet ribosomes. The pellet was resuspended in buffer 2 and layered over a 10 to 40% (w/v) sucrose gradient (buffer 2) and centrifuged at 70,000*g* for 12 h. The gradients were then fractionated using a Brandel gradient fractionator monitoring absorbance at 254 nm, and fractions corresponding to the 70S peak were pooled and concentrated to 11 μM *via* pelleting. 70S aliquots were flash frozen and stored at −80 °C.

### IF2 purification

The alpha form of IF2 was purified using the pET24b-IF2-His6 plasmid through overexpression in BL21 (DE3) Gold cells. Cells were grown in a shaking incubator at 37 °C at 230 rpm. IF2 overexpression was induced by the addition of 1 mM IPTG at OD_600_ of 0.6 for 3.5 h. All subsequent centrifugation steps were performed at 4 °C. Flasks were placed on ice for 5 min, then pelleted by centrifugation at 4000*g* for 20 min. Cell pellets were then washed by resuspension in 20 mM Tris-HCl, pH 8.0, and pelleted again by centrifugation. Cells were lysed in 50 mM Tris-HCl pH 7.5, 60 mM NH_4_Cl, 0.7 mM MgCl_2_, 5 mM imidazole, 15% glycerol, 10 μM GDP, and 2 mM DTT using three passes through the Emulsiflex-C5 high-pressure homogenizer (Avestin). The lysate is then clarified by centrifugation at 16,600*g* for 30 min. The supernatant is loaded onto a 5 ml HisTrap HP column (Cytiva) using an ÄKTA FPLC system. IF2 was eluted using a 5 to 500 mM imidazole gradient and dialyzed into 50 mM Tris-HCl pH 7.5, 60 mM NH_4_Cl, 0.7 mM MgCl_2_, 15% glycerol, 5 μM GDP, and 2 mM DTT. IF2 purity was estimated by SDS-PAGE and its concentration determined using a Bradford assay. Samples were aliquoted and flash frozen in liquid nitrogen to be stored at −80 °C.

### tRNA^fMet2^ M1 purification and aminoacylation

tRNA^fMet2^ with the M1 mutations (G30C, C40G) were cloned and purified at previously described (21). The mutations were introduced into pUC13-trnfM by QuikChange mutagenesis and overexpressed in *E. coli* B105 cells grown in LB with 100 μg/ml ampicillin at 37 °C. Cells were pelleted and washed with PBS (10 mM phosphate pH 7.4, 137 mM NaCl, 3 mM KCl), then resuspended in 20 mM Tris-HCl pH 7.6, 20 mM MgCl_2_. The lysate was phenol extracted using phenol saturated with 25 mM NaOAc pH 5.2, 50 mM NaCl, then 3 M NaOAc pH 5.2 (1/10 volume) and isopropanol (equal volume) were added and incubated for 2 h at 4 °C to precipitate crude tRNA. RNA was pelleted by centrifugation at 8500 rpm for 30 min at 4 °C in a tabletop microfuge and washed with 70% ethanol, dissolved in 200 mM Tris-acetate pH 9.0, and incubated at 37 °C for 30 min, followed by another round of ethanol precipitation. The resulting RNA pellet is then dissolved in water, mixed with loading dye (50% glycerol with bromophenol blue), and tRNA^fMet2^ M1 was separated on a 12% native PAGE gel overnight at 80 V until dye reaches the bottom of the gel. The band corresponding to tRNA^fMet2^ was identified by UV shadowing and excised from the gel. The RNA was extracted using the crush-and-soak method, and then eluted in 300 mM NaOAc pH 5.2, 0.1% SDS, 1 mM EDTA overnight at 4 °C. The eluate solution was then extracted with water-saturated phenol followed with chloroform-isoamyl alcohol (24:1). tRNA^fMet2^ M1 was precipitated overnight at −20 °C by the addition of 3 M NaOAc pH 5.2 (1/10 volume) and ice-cold ethanol (3× volume). The final tRNA^fMet2^ M1 pellet was obtained by centrifugation at 8800 rpm for 30 min at 4 °C in a microfuge, washed with 70% ethanol, dissolved in water, and stored at −80 °C. RNA concentration was determined using absorbance at 260 nm. Aminoacylation by methionyl-tRNA synthetase (MetRS) and formylation by methionyl-tRNA formyltransferase to generate fMet-tRNA^fMet2^ M1 was performed as previously described (40). MetRS and methionyl-tRNA formyltransferase activity were confirmed by acid gel electrophoresis stained with methylene blue and TLC using [^35^S]-methionine as previously described (40).

### 70S complex assembly

70S initiation complexes (ICs) were prepared by stepwise 5 min incubations of 1.6 μM *E coli* 70S, 3.2 μM mRNA (IDT, Table S6), and 4.8 μM fMet-tRNA^fMet2^ M1 in buffer 2 (5 mM HEPES-KOH pH 7.5, 50 mM KCl, 10 mM NH_4_Cl, 6 mM β-Me) at 37 °C. The mRNA sequence is 5′- GGC AAG GAA AUA AAA NUG GUA UAC UUU -3' (“N” of the P-site codon can be A, U, G, or C). For the CUG start codon complex, 6.4 μM tRNA^IleX^ was additionally added to the reaction to monitor for presentation of a +2 frame AUA codon at the ribosomal A site. For the complex containing a CUG start codon, IF2, and the non-hydrolyzable GTP analog GDPCP, a 70S initiation complex was prepared similarly (without tRNA^IleX^), and IF2 was incubated for 20 min with GDPCP in a 1:100 M ratio before being added to the 70S IC (10 μM IF2 and 1 mM GDPCP final concentrations). Following assembly, 70S IC samples were briefly placed on ice until preparing grids for cryo-EM.

### Cryo-EM sample preparation and data collection

To prepare samples for cryo-EM, C-Flat holey carbon gold grids (R1.2/1.3, 300 mesh) were glow discharged in a PELCO easiGlow glow discharger (Ted Pella) for 30 s. Grids were prepared in a Vitrobot Mark IV (FEI) at 4 °C and 100% humidity. 3 μl of the prepared 70S IC sample was applied to grids and allowed to stand for 15 s before blotting and plunging into liquid ethane to vitrify.

### Cryo-EM processing and model building

*E. coli 70S-NUG mRNA-fMet-tRNA*^*fMet*^*(no IF2) structures* (Figs. S1–S3). Prior to processing cryo-EM data, movie frames were aligned using Patch Motion Correction and contrast transfer function (CTF) parameters were estimated using Patch CTF Estimation in cryoSPARC 4.5.3 (41). Micrographs displaying poorer than 5 Å estimated maximum resolution were discarded. Following pre-processing, an initial round of automated particle picking was performed on a random subset of 100 micrographs using the reference-free Blob Picker in cryoSPARC 4.5.3. Extracted particles were subjected to reference-free two-dimensional classification to generate a set of 2D reference images, which were then used as templates to repick particles from the 100 micrograph subset. Template-picked particles were extracted and 2D-classified, and then ribosome-like classes were selected to generate an optimized particle set to train a Topaz particle-picking model (42). The trained Topaz model was used to pick particles from all retained micrographs. Picked particles were extracted with 4× binning and 2D-classified and ribosome-like 2D classes were selected. Three *ab initio* 3D reconstructions (70S ribosomes, 50S subunits, and junk) were generated from selected particles and used as references for heterogeneous 3D refinement, with 70S ribosome particles being selected for further processing and 50S subunit and junk particles being discarded. Selected 70S particles were re-extracted without binning and subjected to initial homogeneous 3D refinement and CTF refinement to correct for microscope aberrations and per-particle defocus estimation. Particles were then subjected to two rounds of iterative reference-based motion correction (43), homogeneous 3D refinement, and CTF refinement (44) to generate an optimized high-resolution particle set. Focused 3D classification was then performed using a mask of the ribosomal A, P, and E sites generated from the A-, P-, and E-site tRNA chains of PDB code 5JTE (45) in UCSF ChimeraX (46). Particles containing P-site tRNAs and either no (UUG, GUG, and AUG mRNA datasets) or minimal (CUG mRNA dataset) E-site tRNA density were subjected to final homogeneous 3D refinement to generate high-resolution 3D reconstructions. The resultant maps were sharpened for modeling using sharpening B-factors automatically estimated from the Guinier plots for each final homogeneous refinement job. A local resolution map was generated for each reconstruction using cryoSPARC’s local resolution estimation implementation with a local FSC cutoff of 0.5.

*E. coli 70S-CUG mRNA-fMet-tRNA*^*fMet*^*-IF2 structure* (Figs. S8 and S9). Micrographs were preprocessed in RELION-3.1 (47) using RELION’s motion correction implementation and CTFFIND-4 for CTF estimation (48). Micrographs displaying poorer than 8 Å estimated maximum resolution were discarded and following pre-processing, particles were picked using the RELION-3.1 Laplacian-of-Gaussian and template-based autopickers (similar to the prior datasets) to generate an optimized particle set for Topaz model training (42). Topaz was then trained and used to pick particles from all retained micrographs, which were extracted with 4× binning, 2D classified, used to generate an initial 3D reconstruction with the stochastic gradient descent method (41), and 3D classified for selection of 70S ribosome particles. Selected particles were re-extracted without binning, subjected to initial 3D auto-refinement, and 3D classified without alignment using a focus mask of the ribosomal A and P sites and GTPase center generated from the tRNA and IF2 chains of PDB code 6O9K (49) in UCSF ChimeraX (46). Particles containing P-site fMet-tRNA^fMet^ and IF2 were subjected to CTF refinement (44, 47) and 3D auto-refinement. Particles were sorted by E-site tRNA content *via* two additional rounds of focused classification, first using a mask generated from the P- and E-site tRNA chains of PDB code 5JTE and then using a mask generated from the E-site tRNA chain of PDB code 5JTE alone (45). Particles containing P- and E-site tRNAs and IF2 were subjected to two rounds of iterative CTF refinement, Bayesian polishing (43), and 3D auto-refinement, then post-processed without sharpening using a solvent mask generated from a 10 Å lowpass filtered copy of the 3D refinement map to yield a final 2.6 Å reconstruction. The resultant map was sharpened for modeling using the Autosharpen tool in PHENIX (50). A local resolution map was generated using RELION’s local resolution estimation implementation.

To build a model of 70S-IC containing fMet-tRNA^fMet^ M1, UCSF ChimeraX was used to rigidly dock coordinates from an existing *E. coli* 70S structure containing P-site fMet-tRNA^fMet^ (PDB code 7K00) (31) into the final 3D refinement map. For the 70S-IC containing IF2 and fMet-tRNA^fMet^ M1, an existing IF2-containing *E. coli* 70S-IC structure was rigidly docked in UCSF ChimeraX (PDB code 6O9K) (49) into the final 3D refinement map. Ligand coordinates and restraints for GDPCP were generated using eLBOW in PHENIX (51) before manually fitting GDPCP coordinates into the nucleotide-binding site of IF2 using COOT (52). PDB code 7K00 was used to obtain initial A-site GUA codon coordinates and initial magnesium ion coordinates for all structures (31). The docked coordinates were subjected to real space refinement in PHENIX using the final sharpened maps and validated using MolProbity (53, 54).

## Data availability

Atomic coordinates and cryo-EM maps have been deposited in the Protein Data Bank, www.pdb.org and Electron Microscopy Data Bank (EMDB): (PDB codes 9AX7, 9AX8, 9CG5, 9CG6, 9CG7; EMDB codes EMD-43929, EMD-43930, EMD-45569, EMD-45572, EMD-45573).

## Supporting information

This article contains supporting information.

## Conflicts of interest

The authors declare that they have no conflicts of interest regarding the contents of this article.
