# Supplementary material for: Structural analysis of noncanonical translation initiation complexes
Source: J Biol Chem. 2024 Sep 1;300(10):107743. doi: 10.1016/j.jbc.2024.107743 (PMC11497404; doi:10.1016/j.jbc.2024.107743)
Supplement: Supporting Figures and Tables [file mmc1.pdf]

Structural analysis of noncanonical translation initiation complexes

Jacob M. Mattingly[1,2], Ha An Nguyen[1], Bappaditya Roy[3], Kurt Fredrick[3] and Christine M. Dunham[1,*]

[1]Department of Chemistry, Emory University, Atlanta, GA, USA
[2]Graduate Program in Biochemistry, Cell and Developmental Biology, Emory University, Atlanta, GA, USA
[3]Department of Microbiology and Center for RNA Biology, The Ohio State University, Columbus, Ohio, USA

SUPPLEMENTARY DATA FILE

Figures S1-S11

Tables S1-S6

Data deposition: Atomic coordinates, and structure factors have been deposited in the Protein Data Bank, www.pdb.org and Electron Microscopy Data Bank (EMDB): (PDB codes 9AX7, 9AX8, 9CG5, 9CG6, 9CG7; EMDB codes EMD-43929, EMD-43930, EMD-45569, EMD-45572, EMD-45573)

Key words: protein synthesis**,** translation initiation, $tRNA^{fMet}$, A-minor motif, 16S rRNA

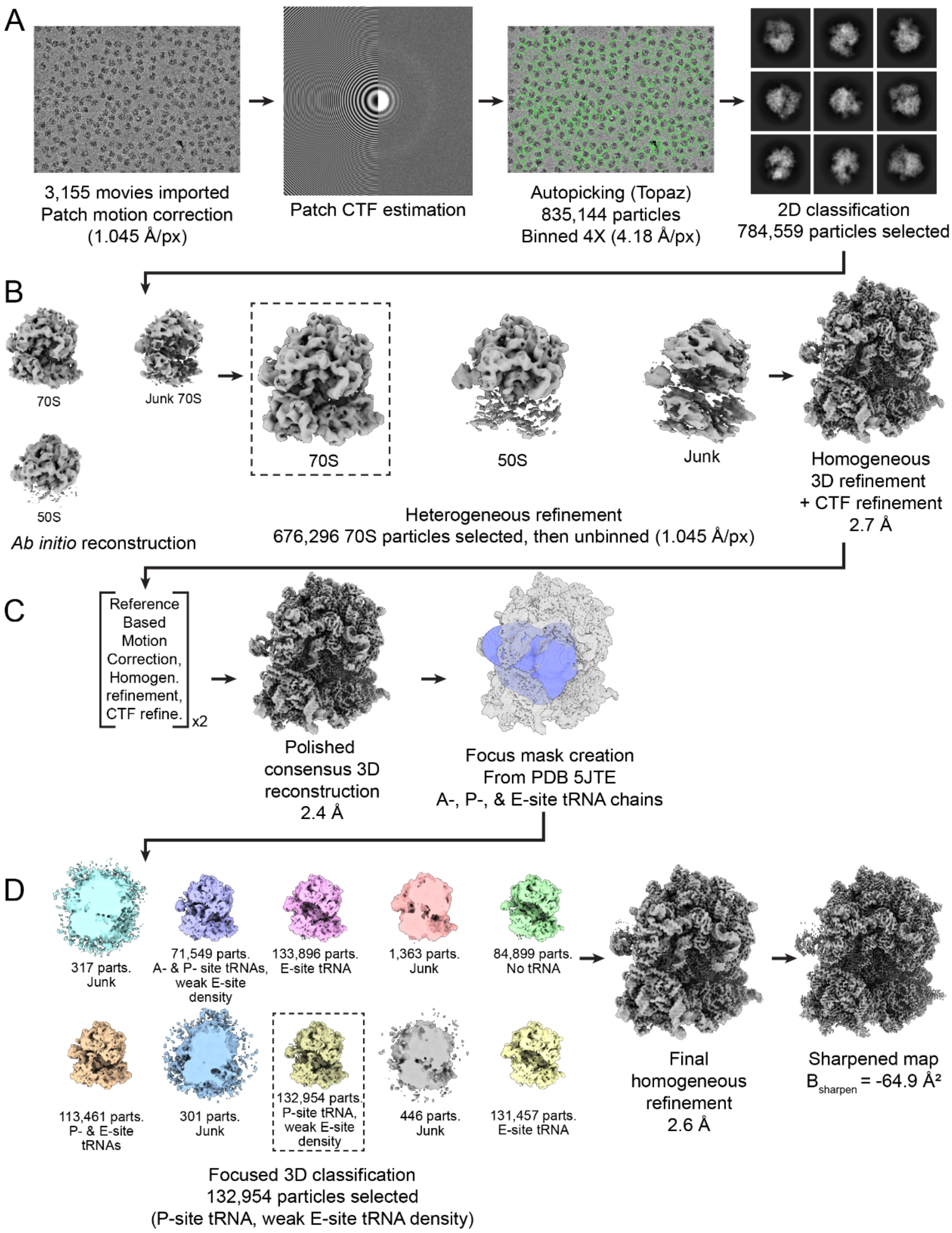

Fig S1. Cryo-EM data processing pipeline (*E. coli* 70S + $tRNA^{fMet}$ M1 + CUG mRNA, no IF2). A. Micrograph preprocessing, particle picking, and reference-free two-dimensional classification. B. *Ab initio* 3D reconstruction of 2D-classified ribosome-like particles, heterogeneous refinement, and initial 3D refinement of selected 70S ribosome particles. C. Iterative reference-based motion correction, homogeneous refinement, and CTF refinement steps to yield a resolution-optimized particle set. Focus mask creation (covering the ribosomal A, P, and E sites) D. Focused 3D classification followed by final homogeneous refinement of selected particles with P-site tRNA and minimal E-site density and B-factor sharpening of the resultant map.

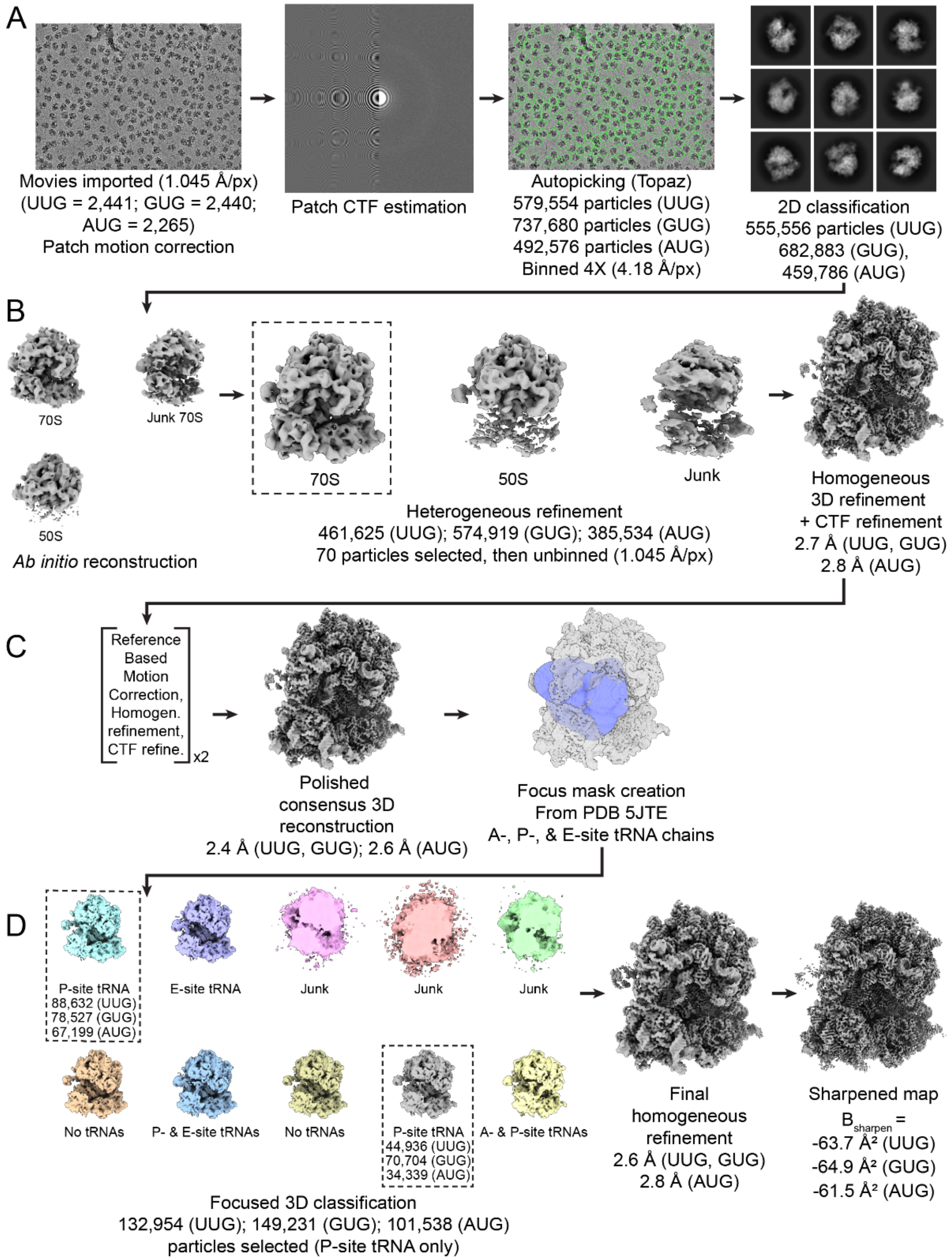

Fig. S2. Cryo-EM data processing pipeline (*E. coli* 70S + tRNA^{fMet} M1 + UUG/GUG/AUG mRNA, no IF2 datasets; UUG dataset shown as examples). A. Micrograph preprocessing, particle picking, and reference-free two-dimensional classification. B. *Ab initio* 3D reconstruction of 2D-classified ribosome-like particles, heterogeneous refinement, and initial 3D refinement of selected 70S ribosome particles. C. Iterative reference-based motion correction, homogeneous refinement, and CTF refinement steps to yield a resolution-optimized particle set. Focus mask creation (covering the ribosomal A, P, and E sites) D. Focused 3D classification followed by final homogeneous refinement of selected particles with only P-site tRNA and B-factor sharpening of the resultant map.

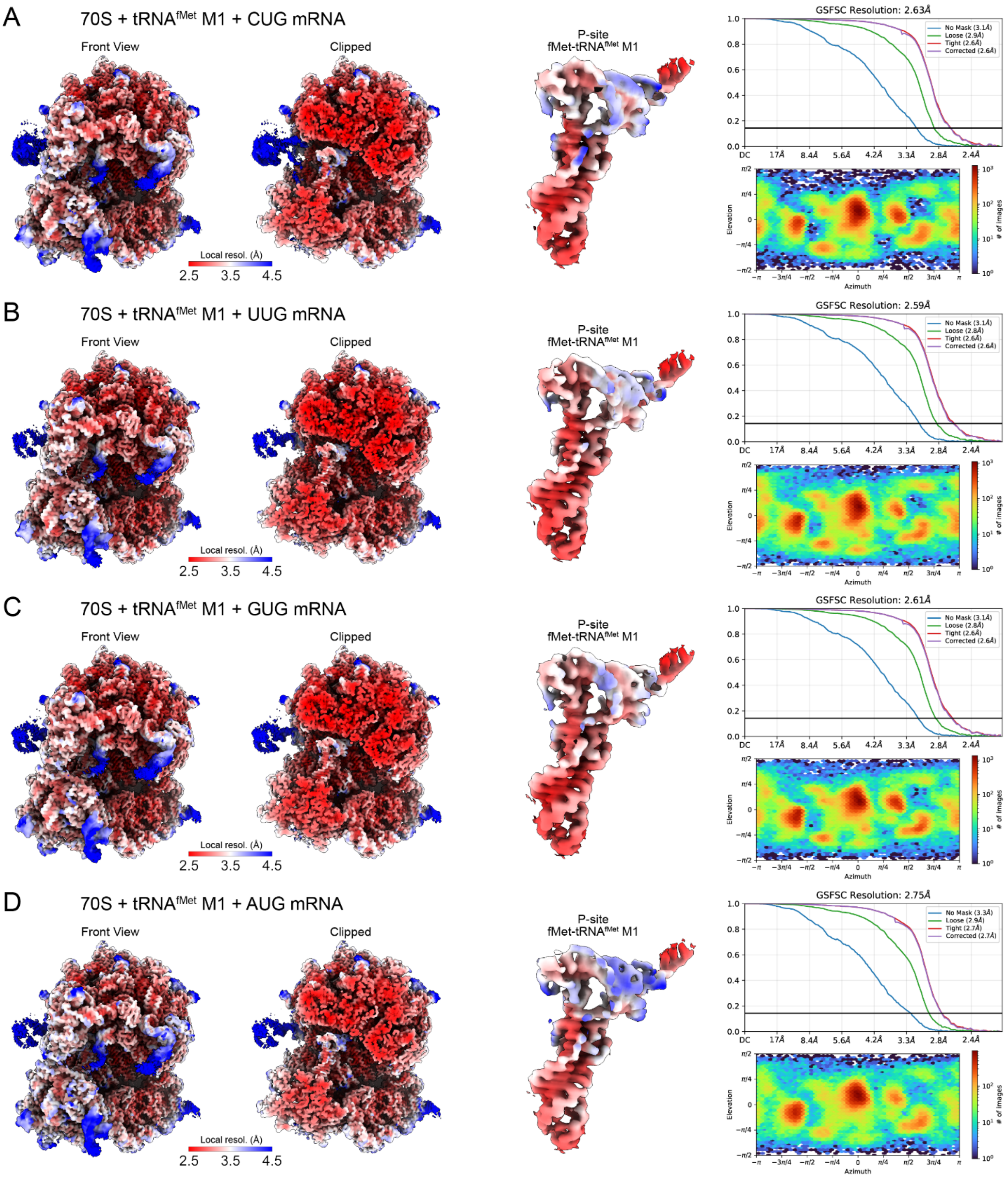

Fig. S3. Data quality metrics (*E. coli* 70S + tRNA^{fMet} M1, no IF2 datasets). A. Data quality metrics for CUG start codon dataset; local resolution maps correspond to 70S front view (left), clipped view for visibility of tRNA binding sites (middle), and zoomed view of P-site fMet-tRNA^{fMet} M1 (right); Fourier shell correlation plot (upper) and orientation distribution plot (lower) from final homogeneous 3D refinement. B. Data quality metrics for UUG start codon dataset. C. Data quality metrics for GUG start codon dataset. D. Data quality metrics for AUG start codon dataset.

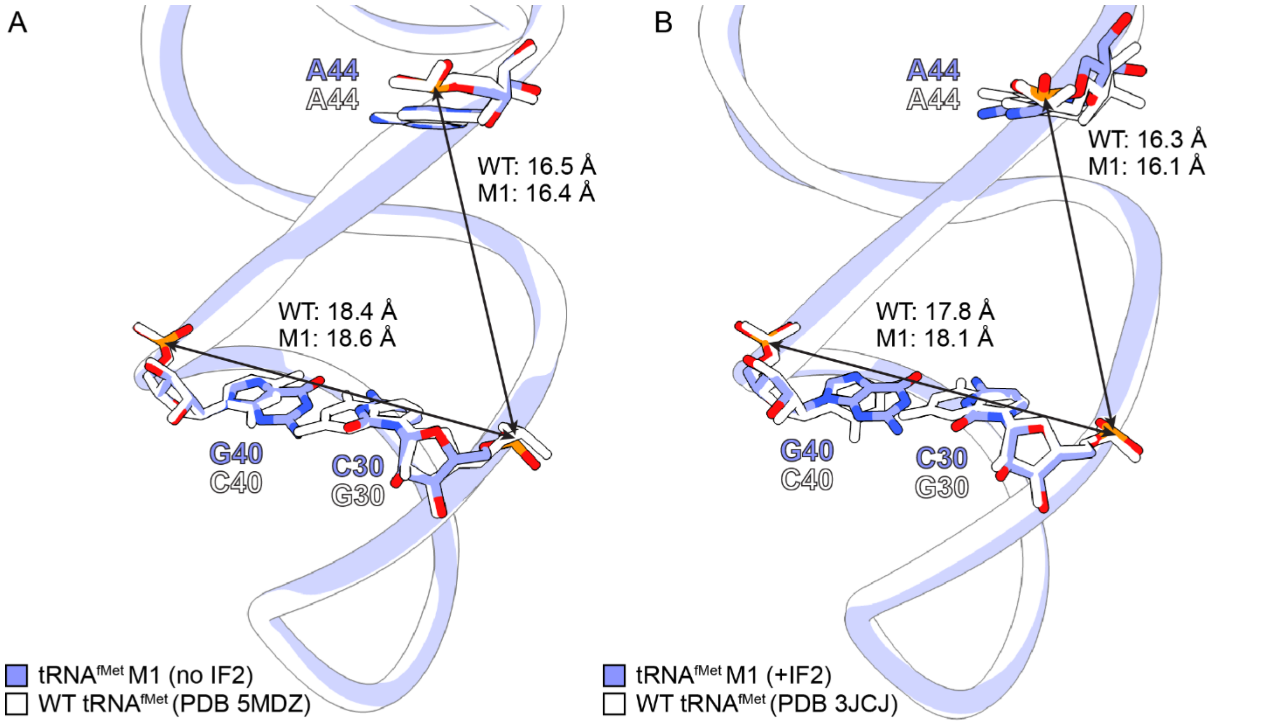

Fig. S4. The M1 mutation does not alter tRNA^{fMet} ASL width or pitch. A. tRNA^{fMet} M1 from 70S ICs prepared without IF2 and WT tRNA^{fMet} from PDB 5MDZ with phosphorus-phosphorus distances measured between nucleotides 30 and 40 (ASL width) and nucleotides 30 and 44 (ASL pitch). B. A. tRNA^{fMet} M1 from 70S ICs containing IF2·GDPCP and WT tRNA^{fMet} from PDB 3JCJ with phosphorus-phosphorus distances measured between nucleotides 30 and 40 (ASL width) and nucleotides 30 and 44 (ASL pitch).

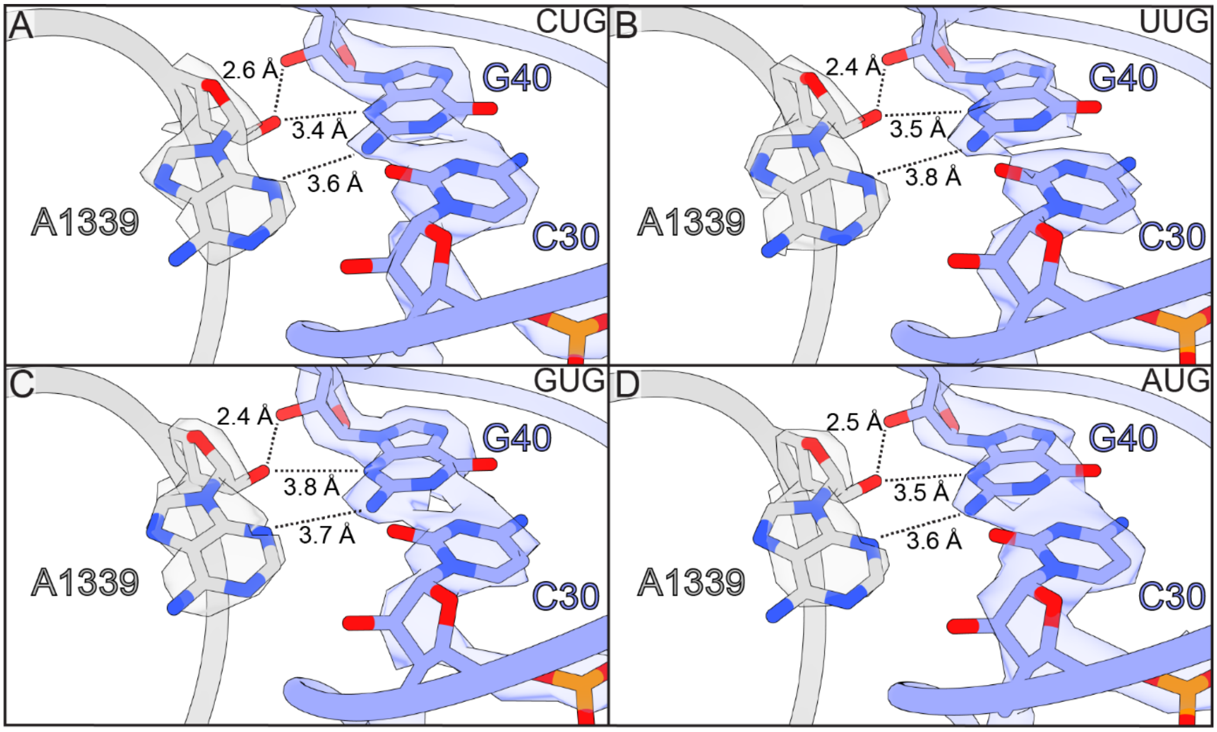

Fig. S5. A-minor interactions between 16S rRNA nucleotide A1339 and the tRNA^{fMet} M1 30-40 base pair are similar in the presence of all NUG start codons. A. A-minor interaction of A1339 with the tRNA^{fMet} M1 30-40 base pair (CUG start codon, map threshold 0.26, map value range -0.877 to 1.41). B. A-minor interaction of A1339 with the tRNA^{fMet} M1 30-40 base pair (UUG start codon, map threshold 0.26, map value range -0.785 to 1.38). C. A-minor interaction of A1339 with the tRNA^{fMet} M1 30-40 base pair (GUG start codon, map threshold 0.26, map value range -0.745 to 1.33). D. A-minor interaction of A1339 with the tRNA^{fMet} M1 30-40 base pair (AUG start codon, map threshold 0.20, map value range -0.527 to 1.03).

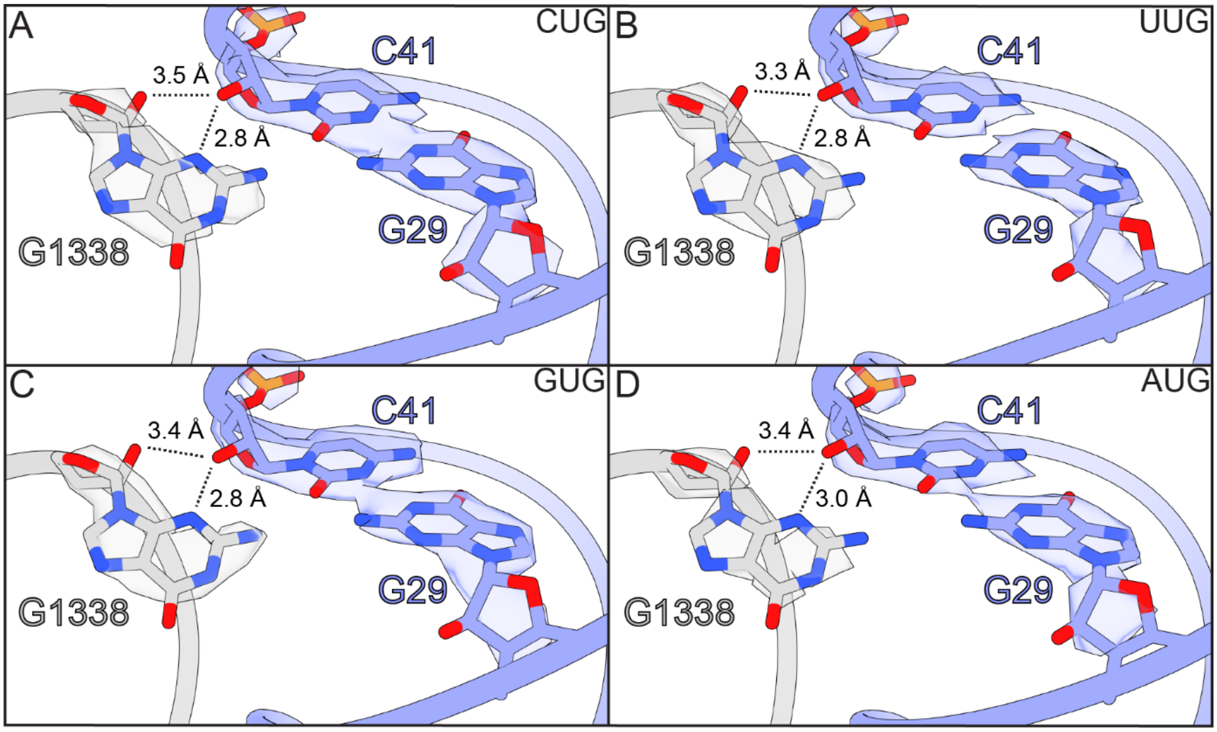

Fig. S6. A-minor interactions between 16S rRNA nucleotide G1338 and tRNA^{fMet} M1 nucleotide C41 are similar in the presence of all NUG start codons. A. A-minor interaction of G1338 with tRNA^{fMet} M1 nucleotide C41 (CUG start codon, map threshold 0.28, map value range -0.877 to 1.41). B. A-minor interaction of G1338 with tRNA^{fMet} M1 nucleotide C41 (UUG start codon, map threshold 0.26, map value range -0.785 to 1.38). C. A-minor interaction of G1338 with tRNA^{fMet} M1 nucleotide C41 (GUG start codon, map threshold 0.26, map value range -0.745 to 1.33). D. A-minor interaction of G1338 with tRNA^{fMet} M1 nucleotide C41 (AUG start codon, map threshold 0.22, map value range -0.527 to 1.03).

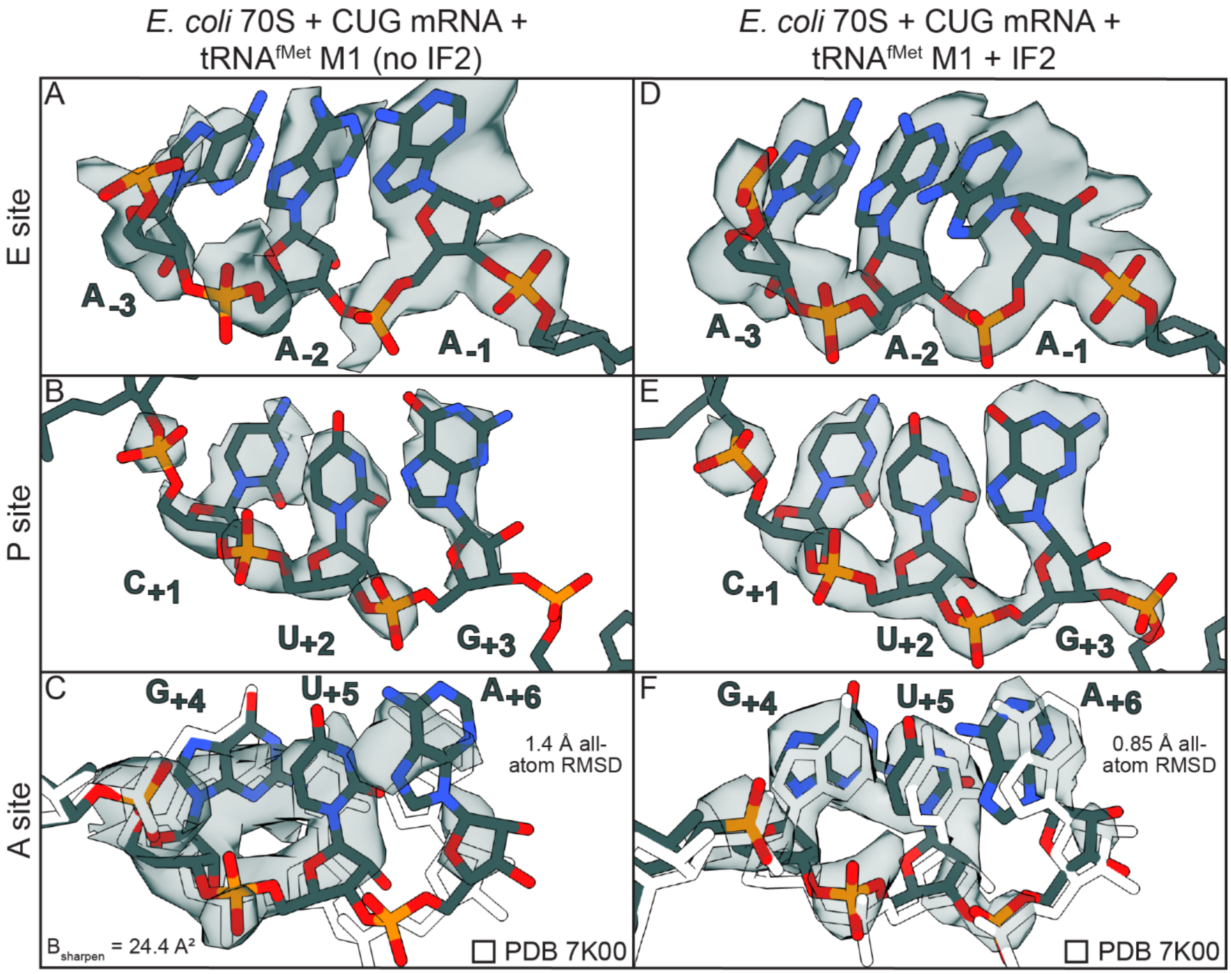

Fig. S7. 70S complexes are observed in the 0 reading frame with functionally presented A-site mRNA codons. A. E-site mRNA codon map and model (no-IF2, CUG start codon structure; map threshold 0.13, map value range -0.877 to 1.41). B. P-site mRNA codon map and model (no-IF2, CUG start codon structure; map threshold 0.3). C. A-site mRNA codon model and alternative sharpened map generated using PHENIX Autosharpen (no-IF2, CUG start codon structure with A-site codon from PDB 7K00 superimposed; global sharpening B-factor of 24.41 $Å^2$, map threshold 2.9, map value range -13.7 to 29.8). Models were aligned on their 16S rRNA platform domains for measurement of an all-atom RMSD for their A-site codons. D. E-site mRNA codon map and model (+IF2 structure; map threshold 2.9, map value range -11.3 to 28.2). E . P-site mRNA codon map and model (+IF2 structure; map threshold 4.5).). F. A-site mRNA codon map and model (+IF2, CUG start codon structure A-site mRNA codon from PDB 7K00 superimposed; map threshold 2.4). Models were aligned on their 16S rRNA platform domains for measurement of an all-atom RMSD for their A-site codons.

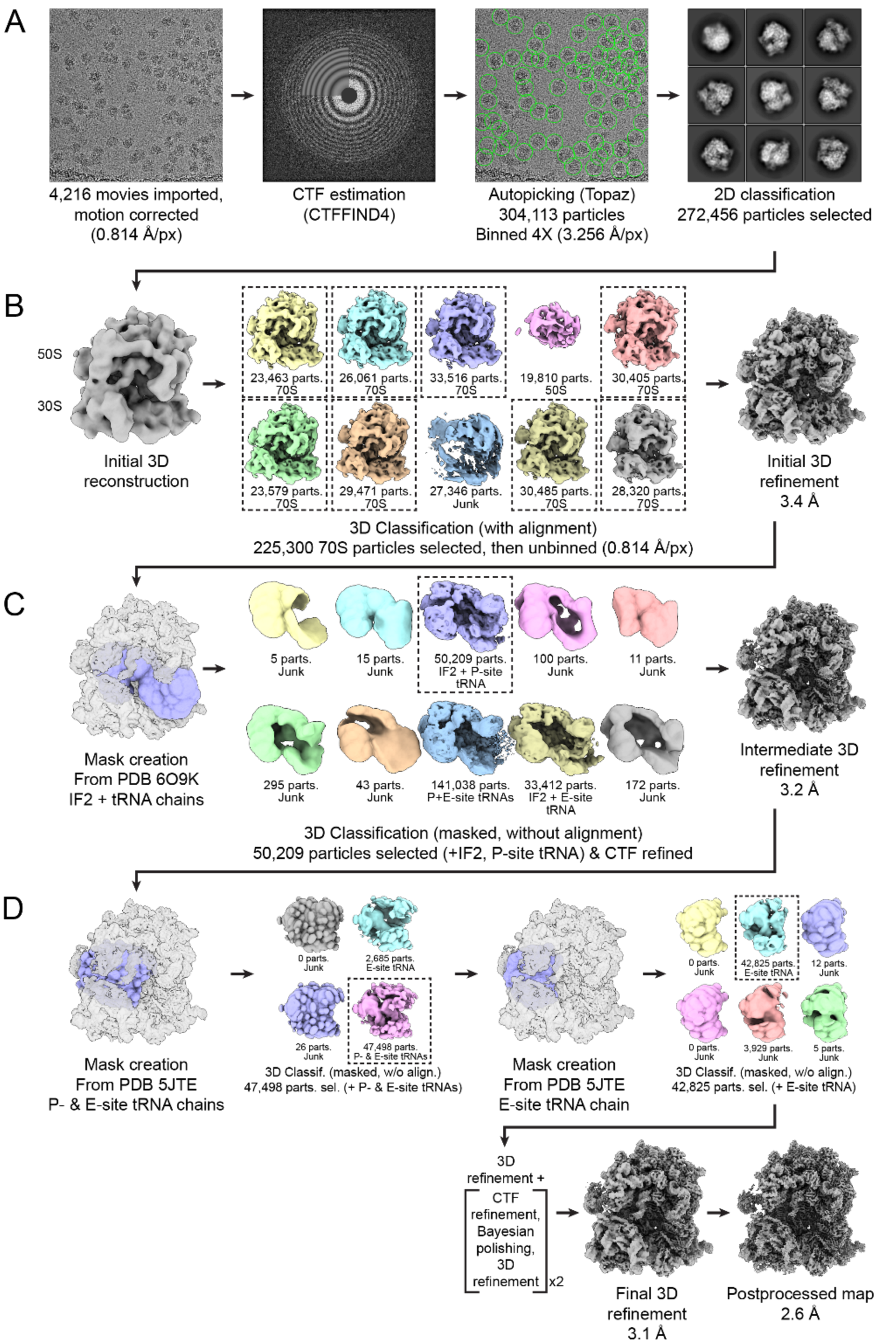

Fig. S8. Cryo-EM data processing pipeline (*E. coli* 70S + tRNA^{fMet} M1 + IF2). A. Micrograph preprocessing, particle picking, and reference-free two-dimensional classification. B. Initial 3D reconstruction of 2D-classified ribosome-like particles, three-dimensional classification with particle alignment, and initial 3D refinement of selected 70S ribosome particles. C. Mask creation (covering the IF2 binding site and ribosomal P site), followed by focused three-dimensional classification without particle alignment and 3D refinement of selected IF2-containing particles. D. Mask creation (covering the ribosomal P and E sites), focused 3D classification without alignment, additional focused classification with a mask covering the ribosomal E site, and final CTF refinement, Bayesian particle polishing, 3D refinement, and postprocessing steps to yield a 2.6 Å 3D reconstruction.

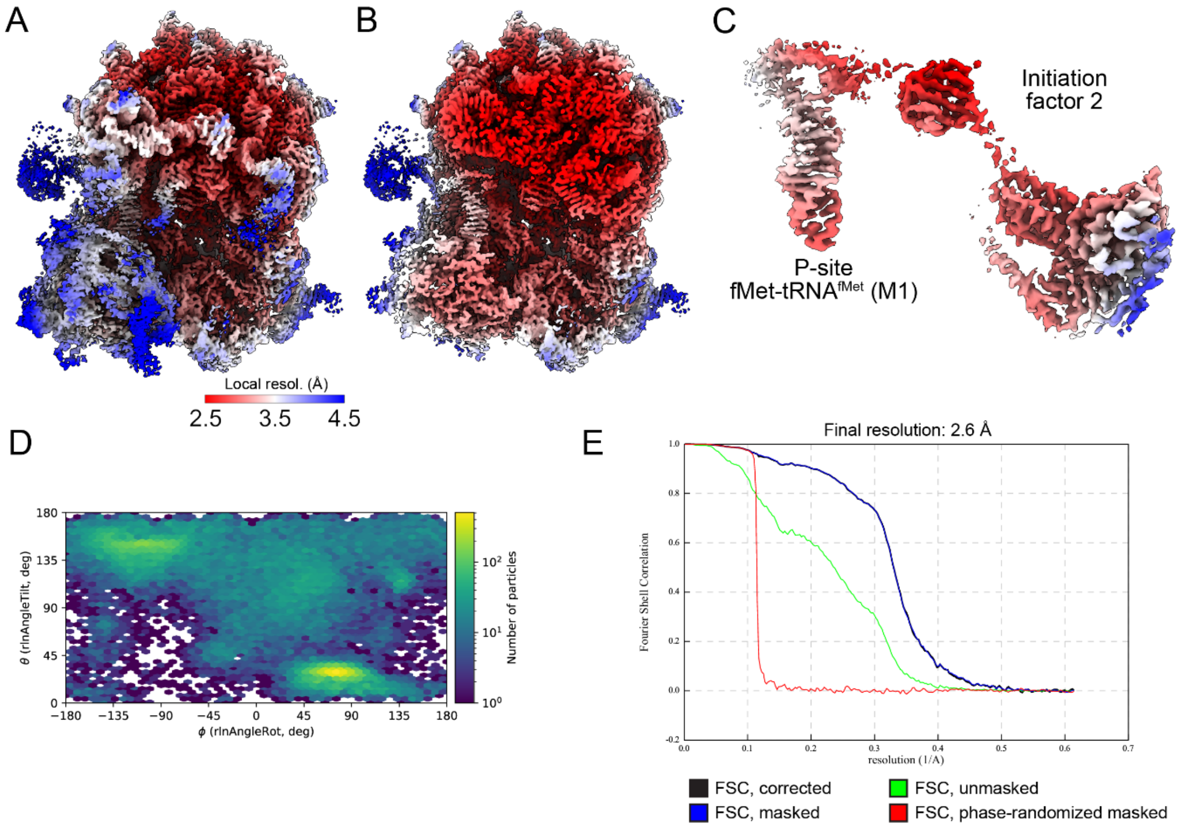

Fig. S9. Data quality metrics (*E. coli* 70S + tRNA^{fMet} M1 + IF2). A. Local resolution map (external view) generated using RELION-3.1's local resolution estimation implementation. B. Local resolution map with clipping plane applied for visibility of bound P-site fMet-tRNA^{fMet} M1 and IF2. C. Local resolution of fMet-tRNA^{fMet} M1 and IF2 map fragments extracted from final 3D reconstruction displayed in (A). D. Angular distribution plot of 42,825 IF2-containing particles generated using AngDist. E. Fourier shell correlation plot from RELION-3.1 postprocessing job yielding a resolution of 2.6 Å at the FSC=0.143 cutoff upon application of a solvent mask.

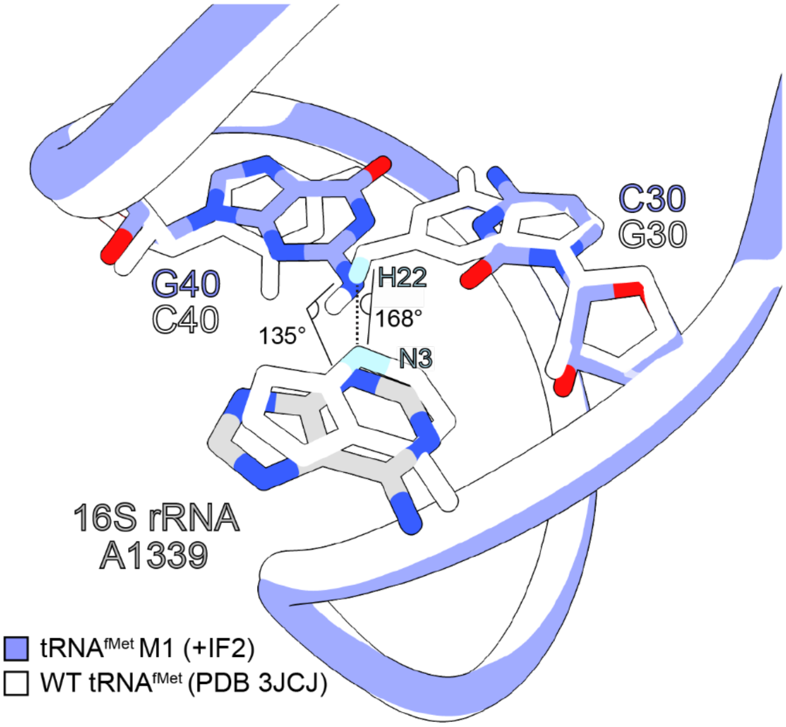

Fig. S10. The M1 mutation appears to weaken tRNA^{fMet} interaction with 16S nucleotide A1339 by reducing hydrogen bonding angles with the 30-40 base pair. IF2 binding to complexes containing tRNA^{fMet} M1 does not improve the linearity of hydrogen bonding between A1339 and the 30-40 base pair.

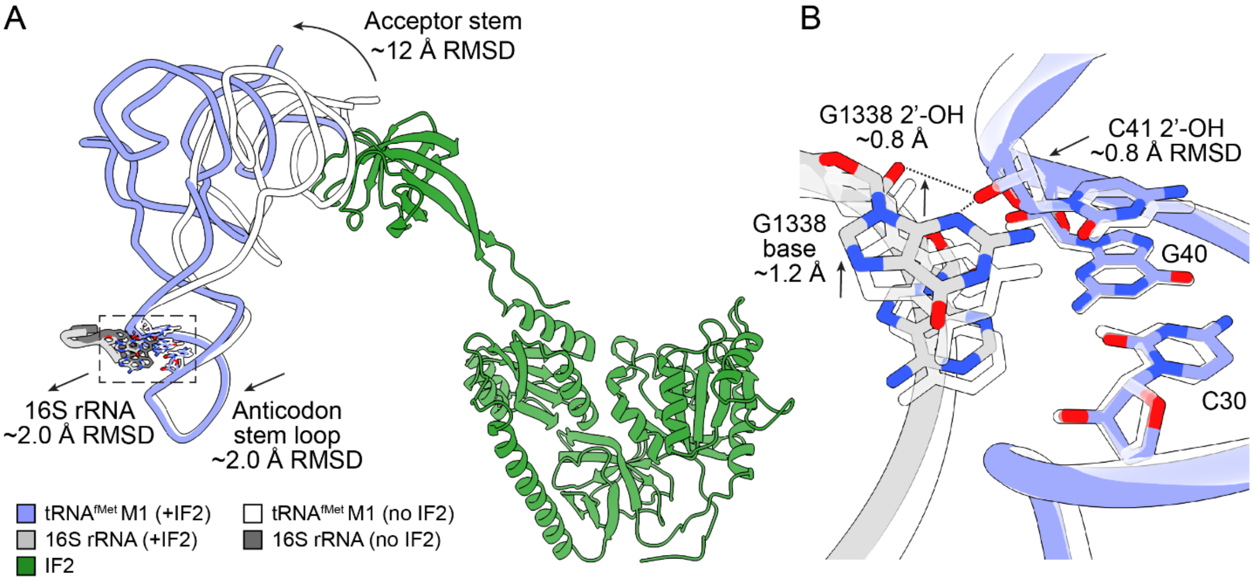

Fig. S11. IF2 improves the interaction between 16S rRNA and the tRNA^{fMet} M1 minor groove. A. When IF2 is present, the acceptor stem of tRNA^{fMet} M1 is displaced ~12 Å toward the ribosomal E site while the anticodon stem loop is displaced roughly 2 Å toward the ribosomal 30S head domain. The single-stranded 16S rRNA loop containing NTs 1335-1339 is displaced roughly 2 Å along with the tRNA^{fMet} M1 ASL when IF2 is present (structures aligned on the 16S rRNA platform domain). B. The interaction of IF2 with tRNA^{fMet} M1 during initiation displaces the 2'-OH of ASL nucleotide C41 roughly 0.8 Å toward the 16S rRNA, the 16S nucleotide G1338 base roughly 1.2 Å upward along the tRNA minor groove, and G1338 2'-OH 0.8 Å upward along the minor groove relative to the C30-G40 base pair, slightly strengthening tRNA gripping by the ribosomal P site (structures aligned on the tRNA^{fMet} M1 C30-G40 base pair).

Table S1. Cryo-EM data collection and model statistics for the *E. coli* 70S + $tRNA^{fMet}$ M1 + CUG start codon dataset (no IF2).

EMDB accession	EMD-43929	
PDB ID	9AX7	
Name	70S IC ($tRNA^{fMet}$ M1 + CUG start codon)	
**Data collection**		
Microscope	FEI Talos Arctica	
Detector	Gatan K3 Bioquantum	
Voltage (keV)	200	
Electron exposure ($e^-/Å^2$)	58.4	
Pixel size (Å)	1.045	
Defocus range (µm)	-0.6-1.8	
Frames per movie	40	
Micrographs (#)	3,155	
Initial particles (#)	835,144	
Final particles (#)	132,954	
**Model refinement and validation statistics**		
**Composition (#)**		
Atoms	140,102	
Residues	Protein: 5,585; Nucleotide: 4,472	
Ligands	Mg: 394<br>Zn: 2	
**Bonds (RMSD)**		
Length (Å) (# > 4σ)	0.003 (0)	
Angles (°) (# > 4σ)	0.651 (2)	
MolProbity score	1.59	
Clash score	7.43	
**Ramachandran plot (%)**		
Outliers	0.04	
Allowed	3.00	
Favored	96.97	
Rotamer outliers (%)	0.28	
Cβ outliers (%)	0.00	
**Peptide plane (%)**		
Cis proline/general	2.2/0.0	
Twisted proline/general	0.0/0.0	
CaBLAM outliers (%)	1.80	
**ADP (B-factors)**	min/max/mean	
Protein	0.00/109.35/43.20	
Nucleotide	0.00/152.32/34.10	
Ligand	0.22/82.03/24.98	
**Resolution Estimates (Å)**	Masked	Unmasked
d FSC (half maps; 0.143)	2.8	2.9
d 99 (full/half1/half2)	2.9/2.1/2.1	2.8/2.1/2.1
d model	2.9	2.9
d FSC model (0/0.143/0.5)	2.6/2.6/2.8	2.6/2.6/3.0
Map min/max/mean	-0.88/1.41/0.01	
**Model vs. Data**		
CC (mask)	0.85	

Table S2. Cryo-EM data collection and model statistics for the *E. coli* 70S + $tRNA^{fMet}$ M1 + UUG start codon dataset.

**EMDB accession**	**EMD-45569**	
**PDB ID**	**9CG5**	
**Name**	**70S IC ($tRNA^{fMet}$ M1 + UUG start codon)**	
**Data collection**		
Microscope	FEI Talos Arctica	
Detector	Gatan K3 Bioquantum	
Voltage (keV)	200	
Electron exposure ($e^-/Å^2$)	58.4	
Pixel size (Å)	1.045	
Defocus range (µm)	-0.6-1.8	
Frames per movie	40	
Micrographs (#)	2,441	
Initial particles (#)	579,544	
Final particles (#)	133,568	
**Model refinement and validation statistics**		
**Composition (#)**		
Atoms	140,102	
Residues	Protein: 5,585; Nucleotide: 4,472	
Ligands	Mg: 309<br>Zn: 2	
**Bonds (RMSD)**		
Length (Å) (# > 4σ)	0.008 (2)	
Angles (°) (# > 4σ)	0.834 (4)	
MolProbity score	1.66	
Clash score	7.00	
**Ramachandran plot (%)**		
Outliers	0.11	
Allowed	3.87	
Favored	96.02	
Rotamer outliers (%)	0.94	
Cβ outliers (%)	0.00	
**Peptide plane (%)**		
Cis proline/general	2.2/0.0	
Twisted proline/general	0.0/0.0	
CaBLAM outliers (%)	1.91	
**ADP (B-factors)**	min/max/mean	
Protein	0.00/107.75/39.90	
Nucleotide	0.00/125.66/32.79	
Ligand	0.00/76.48/23.21	
**Resolution Estimates (Å)**	Masked	Unmasked
d FSC (half maps; 0.143)	2.7	2.9
d 99 (full/half1/half2)	2.8/2.1/2.1	2.7/2.1/2.1
d model	2.9	2.9
d FSC model (0/0.143/0.5)	2.6/2.6/2.8	2.6/2.6/2.9
Map min/max/mean	-0.78/1.38/0.01	
**Model vs. Data**		
CC (mask)	0.87	

Table S3. Cryo-EM data collection and model statistics for the *E. coli* 70S + $tRNA^{fMet}$ M1 + GUG start codon dataset.

**EMDB accession**	**EMD-45572**	
**PDB ID**	**9CG6**	
**Name**	**70S IC ($tRNA^{fMet}$ M1 + GUG start codon)**	
**Data collection**		
Microscope	FEI Talos Arctica	
Detector	Gatan K3 Bioquantum	
Voltage (keV)	200	
Electron exposure (e^-/Å^2)	58.4	
Pixel size (Å)	1.045	
Defocus range (µm)	-0.6-1.8	
Frames per movie	40	
Micrographs (#)	2,440	
Initial particles (#)	737,680	
Final particles (#)	149,231	
**Model refinement and validation statistics**		
**Composition (#)**		
Atoms	140,105	
Residues	Protein: 5,585; Nucleotide: 4,447	
Ligands	Mg: 309<br>Zn: 2	
**Bonds (RMSD)**		
Length (Å) (# > 4σ)	0.007 (2)	
Angles (°) (# > 4σ)	0.797 (11)	
MolProbity score	1.64	
Clash score	6.85	
**Ramachandran plot (%)**		
Outliers	0.15	
Allowed	3.73	
Favored	96.13	
Rotamer outliers (%)	1.01	
Cβ outliers (%)	0.00	
**Peptide plane (%)**		
Cis proline/general	2.2/0.0	
Twisted proline/general	0.0/0.0	
CaBLAM outliers (%)	2.08	
**ADP (B-factors)**	min/max/mean	
Protein	0.00/110.95/41.08	
Nucleotide	0.00/123.13/33.55	
Ligand	0.61/78.92/24.25	
**Resolution Estimates (Å)**	Masked	Unmasked
d FSC (half maps; 0.143)	2.7	2.9
d 99 (full/half1/half2)	2.9/2.2/2.2	2.8/2.1/2.1
d model	2.9	2.9
d FSC model (0/0.143/0.5)	2.6/2.6/2.8	2.6/2.6/2.9
Map min/max/mean	-0.74/1.33/0.01	
**Model vs. Data**		
CC (mask)	0.87	

Table S4. Cryo-EM data collection and model statistics for the *E. coli* 70S + tRNA^{fMet} M1 + AUG start codon dataset.

**EMDB accession**	**EMD-45573**	
**PDB ID**	**9CG7**	
**Name**	**70S IC (tRNA^{fMet} M1 + AUG start codon)**	
**Data collection**		
Microscope	FEI Talos Arctica	
Detector	Gatan K3 Bioquantum	
Voltage (keV)	200	
Electron exposure (e$^-$/Å^2)	58.4	
Pixel size (Å)	1.045	
Defocus range (μm)	-0.6-1.8	
Frames per movie	40	
Micrographs (#)	2,265	
Initial particles (#)	492,576	
Final particles (#)	101,538	
**Model refinement and validation statistics**		
**Composition (#)**		
Atoms	140,104	
Residues	Protein: 5,585; Nucleotide: 4,447	
Ligands	Mg: 309<br>Zn: 2	
**Bonds (RMSD)**		
Length (Å) (# > 4σ)	0.007 (7)	
Angles (°) (# > 4σ)	0.776 (4)	
MolProbity score	1.64	
Clash score	6.43	
**Ramachandran plot (%)**		
Outliers	0.11	
Allowed	4.06	
Favored	95.83	
Rotamer outliers (%)	0.37	
Cβ outliers (%)	0.00	
**Peptide plane (%)**		
Cis proline/general	2.2/0.0	
Twisted proline/general	0.0/0.0	
CaBLAM outliers (%)	2.10	
**ADP (B-factors)**	min/max/mean	
Protein	0.00/111.21/43.18	
Nucleotide	0.00/114.69/40.32	
Ligand	4.42/83.88/25.80	
**Resolution Estimates (Å)**	Masked	Unmasked
d FSC (half maps; 0.143)	2.9	3.0
d 99 (full/half1/half2)	3.0/2.1/2.1	2.9/2.1/2.1
d model	3.0	2.9
d FSC model (0/0.143/0.5)	2.7/2.7/2.9	2.7/2.7/3.0
Map min/max/mean	-0.53/1.03/0.01	
**Model vs. Data**		
CC (mask)	0.87	

Table S5. Cryo-EM data collection and model statistics for the *E. coli* 70S + $tRNA^{fMet}$ M1 + IF2·GDPCP + CUG start codon dataset.

**EMDB accession**	**EMD-43930**	
**PDB ID**	**9AX8**	
**Name**	**70S IC ($tRNA^{fMet}$ M1, IF2·GDPCP + CUG start codon)**	
**Data collection**		
Microscope	TFS Krios	
Detector	TFS Falcon 4i	
Voltage (keV)	300	
Electron exposure ($e^-/Å^2$)	50	
Pixel size (Å)	0.814	
Defocus range (μm)	-0.5-2.5	
Frames per movie	29	
Micrographs (#)	4,216	
Initial particles (#)	304,113	
Final particles (#)	42,825	
**Model refinement and validation statistics**		
**Composition (#)**		
Atoms	145,067	
Residues	Protein: 5,904; Nucleotide: 4,597	
Ligands	Mg: 287<br>GDPCP: 1	
**Bonds (RMSD)**		
Length (Å) (# > 4σ)	0.003 (7)	
Angles (°) (# > 4σ)	0.661 (19)	
MolProbity score	2.29	
Clash score	6.90	
**Ramachandran plot (%)**		
Outliers	0.76	
Allowed	7.95	
Favored	91.29	
Rotamer outliers (%)	3.32	
Cβ outliers (%)	0.00	
**Peptide plane (%)**		
Cis proline/general	0.0/0.0	
Twisted proline/general	0.0/0.0	
CaBLAM outliers (%)	5.83	
**ADP (B-factors)**	min/max/mean	
Protein	10.51/182.48/66.46	
Nucleotide	0.00/356.69/80.36	
Ligand	9.69/79.47/34.34	
**Resolution Estimates (Å)**	Masked	Unmasked
d FSC (half maps; 0.143)	2.6	2.9
d 99 (full/half1/half2)	3.1/1.8/1.8	3.0/1.7/1.7
d model	2.9	2.9
d FSC model (0/0.143/0.5)	2.4/2.6/2.9	2.6/2.7/3.0
Map min/max/mean	-11.34/28.17/0.15	
**Model vs. Data**		
CC (mask)	0.86	

Table S6. Sequences of nucleic acids used in this study.

Name	Nucleotide sequence (5' → 3')	Source
mRNA (CUG)	GGCAAGGAAAUAAAACUGGUAUACUUU	Chemically synthesized (IDT)
mRNA (UUG)	GGCAAGGAAAUAAAAUUGGUAUACUUU	Chemically synthesized (IDT)
mRNA (GUG)	GGCAAGGAAAUAAAAGUGGUAUACUUU	Chemically synthesized (IDT)
mRNA (AUG)	GGCAAGGAAAUAAAAAUGGUAUACUUU	Chemically synthesized (IDT)
